# Author Correction: Super-resolution microscopy compatible fluorescent probes reveal endogenous glucagon-like peptide-1 receptor distribution and dynamics

**DOI:** 10.1038/s41467-020-19101-4

**Published:** 2020-10-09

**Authors:** Julia Ast, Anastasia Arvaniti, Nicholas H. F. Fine, Daniela Nasteska, Fiona B. Ashford, Zania Stamataki, Zsombor Koszegi, Andrea Bacon, Ben J. Jones, Maria A. Lucey, Shugo Sasaki, Daniel I. Brierley, Benoit Hastoy, Alejandra Tomas, Giuseppe D’Agostino, Frank Reimann, Francis C. Lynn, Christopher A. Reissaus, Amelia K. Linnemann, Elisa D’Este, Davide Calebiro, Stefan Trapp, Kai Johnsson, Tom Podewin, Johannes Broichhagen, David J. Hodson

**Affiliations:** 1 Institute of Metabolism and Systems Research (IMSR), and Centre of Membrane Proteins and Receptors (COMPARE), University of Birmingham, Birmingham, UK; 2 Centre for Endocrinology, Diabetes and Metabolism, Birmingham Health Partners, Birmingham, UK; 3 Centre for Liver Research, College of Medical and Dental Sciences, Institute for Immunology and Immunotherapy, University of Birmingham, Birmingham, UK; 4 Genome Editing Facility, Technology Hub, University of Birmingham, Birmingham, UK; 5 Division of Diabetes, Endocrinology and Metabolism, Section of Investigative Medicine, Imperial College London, London, UK; 6 Diabetes Research Group, BC Children’s Hospital Research Institute, Vancouver, BC, Canada; Department of Surgery, University of British Columbia, Vancouver, BC Canada; 7 Centre for Cardiovascular and Metabolic Neuroscience, Department of Neuroscience, Physiology & Pharmacology, University College London, London, UK; 8 Oxford Centre for Diabetes, Endocrinology & Metabolism, University of Oxford, Oxford, UK; 9 Division of Diabetes, Endocrinology and Metabolism, Section of Cell Biology and Functional Genomics, Imperial College London, London, UK; 10 Faculty of Biology, Medicine and Health, University of Manchester, Oxford Road, Manchester, UK; 11 Wellcome Trust-MRC Institute of Metabolic Science, University of Cambridge, Cambridge, UK; 12 Department of Pediatrics, Indiana University School of Medicine, Indianapolis, IN USA; 13 Optical Microscopy Facility, Max Planck Institute for Medical Research, Heidelberg, Germany; 14 Department of Chemical Biology, Max Planck Institute for Medical Research, Heidelberg, Germany

**Keywords:** Optical imaging, Super-resolution microscopy, Chemical tools, Endocrine system and metabolic diseases

Correction to: *Nature Communications* 10.1038/s41467-020-14309-w, published online 24 January 2020.

The original version of this Article contained an error in the Methods section, when reporting ethical approval related to the generation of hESCs, which read: ‘hESC (WA01/H1; hPSCreg name WAe001-A) (obtained from WiCell) were generated by the originating institute with informed consent and ethical approval from the Robert-Koch Institut, Berlin (Az.3.04.02/0101) and NIH (NIHhESC-10-0043). Studies with hESC (WA01/H1) were approved by the BC Children’s and Women’s Hospital Human Research Ethics Board (Approval #H09-00676)’. However, the cells were purchased from WiCell (Wisconsin) and the Robert-Koch Institute and NIH were not involved in the study. The sentence has now been corrected to state: ‘hESC (WA01/H1; hPSCreg name WAe001-A) were purchased from WiCell (Wisconsin) (full details including ethics and informed consent available at hPSCreg, cell line WAe001-A). Studies with hESC (WA01/H1) were approved by the BC Children’s and Women’s Hospital Human Research Ethics Board (Approval #H09-00676)’. This has been corrected in both the PDF and HTML versions of the Article.

