# Supplementary material for: Super-resolution microscopy compatible fluorescent probes reveal endogenous glucagon-like peptide-1 receptor distribution and dynamics
Source: Nat Commun. 2020 Jan 24;11:467. doi: 10.1038/s41467-020-14309-w (PMC6981144; doi:10.1038/s41467-020-14309-w)
Supplement: Supplementary file 1 — Supplementary Information [file 41467_2020_14309_MOESM1_ESM.pdf]

SUPPLEMENTARY INFORMATION

Super-resolution microscopy compatible fluorescent probes reveal endogenous glucagon-like peptide-1 receptor distribution and dynamics

Julia Ast[1,2], Anastasia Arvaniti[1,2], Nicholas H.F. Fine[1,2], Daniela Nasteska[1,2], Fiona B. Ashford[1,2], Zania Stamataki[3], Zsombor Koszegi[1,2], Andrea Bacon[4], Ben J. Jones[5], Maria A. Lucey[5], Shugo Sasaki[6], Daniel I. Brierley[7], Benoit Hastoy[8], Alejandra Tomas[9], Giuseppe D'Agostino[10], Frank Reimann[11], Francis C. Lynn[6], Christopher A. Reissaus[12], Amelia K. Linnemann[12], Elisa D'Este[13], Davide Calebiro[1,2], Stefan Trapp[7], Kai Johnsson[14], Tom Podewin[14*], Johannes Broichhagen[14*] and David J. Hodson[1,2*]

[1] Institute of Metabolism and Systems Research (IMSR), and Centre of Membrane Proteins and Receptors (COMPARE), University of Birmingham, Birmingham, UK.
[2] Centre for Endocrinology, Diabetes and Metabolism, Birmingham Health Partners, Birmingham, UK.
[3] Centre for Liver Research, College of Medical and Dental Sciences, Institute for Immunology and Immunotherapy, University of Birmingham, Birmingham, UK.
[4] Genome Editing Facility, Technology Hub, University of Birmingham, Birmingham, UK.
[5] Imperial College London, Section of Investigative Medicine, Division of Diabetes, Endocrinology and Metabolism, London, UK.
[6] Diabetes Research Group, BC Children's Hospital Research Institute, Vancouver, BC, Canada; Department of Surgery, University of British Columbia, Vancouver, BC, Canada.
[7] Centre for Cardiovascular and Metabolic Neuroscience, Department of Neuroscience, Physiology & Pharmacology, University College London, London, UK
[8] Oxford Centre for Diabetes, Endocrinology & Metabolism, University of Oxford, Oxford, UK.
[9] Imperial College London, Section of Cell Biology and Functional Genomics, Division of Diabetes, Endocrinology and Metabolism, London, UK.
[10] Faculty of Biology, Medicine and Health, University of Manchester, Oxford Road, Manchester, UK.
[11] Wellcome Trust-MRC Institute of Metabolic Science, University of Cambridge, Cambridge, U.K
[12] Department of Pediatrics, Indiana University School of Medicine, Indianapolis, IN, USA.
[13] Optical Microscopy Facility, Max Planck Institute for Medical Research, Heidelberg, Germany.
[14] Department of Chemical Biology, Max Planck Institute for Medical Research, Heidelberg, Germany.

*Correspondence should be addressed to: d.hodson@bham.ac.uk, johannes.broichhagen@mr.mpg.de or tom.podewin@mpimf-heidelberg.mpg.de

SUPPLEMENTARY METHODS

General chemistry

Solvents for chromatography and reactions were purchased HPLC grade (Sigma-Aldrich, 99.8%, extra dry over molecular sieves). If necessary, solvents were degassed either by freeze-pump-thaw or by bubbling N_2 through the vigorously stirred solution for several minutes. Unless otherwise stated, all other reagents were used without further purification from commercial sources.

LC-MS was performed on a Shimadzu MS2020 connected to a Nexera UHPLC system equipped with a Waters ACQUITY UPLC BEH C18 (1.7 µm, 50 × 2.1 mm). Buffer A: 0.1% FA in H_2O Buffer B: acetonitrile. The typical gradient was from 10% B for 0.5 min → gradient to 90% B over 4.5 min → 90% B for 0.5 min → gradient to 99% B over 0.5 min with 1 mL/min flow. Retention times (t_R) are given in minutes (min).

Preparative and analytical RP-HPLC was performed on a Waters e2695 system equipped with a 2998 PDA detector for product collection (at 220, 550 or 650 nm) on a Supelco Ascentis® C18 HPLC Column (preparative: 5 µm, 250 × 21.2 mm; analytical: 5 µm, 250 × 10 mm). Buffer A: 0.1% TFA in H_2O Buffer B: acetonitrile. The typical gradient was from 10% B for 5 min → gradient to 90% B over 45 min → 90% B for 5 min → gradient to 99% B over 5 min with 8 mL/min flow (preparative) or 4 mL/min (analytical).

NMR spectra were recorded in deuterated solvents on BRUKER Avance III HD 400 (equipped with a CryoProbe™) and calibrated to residual solvent peaks (^{1}H in ppm): DMSO-d6 (2.50/39.52). Multiplicities are abbreviated as follows: s = singlet, d = doublet, t = triplet, q = quartet, br = broad, m = multiplet. Spectra are reported based on appearance, not on theoretical multiplicities derived from structural information.

High resolution mass spectrometry was performed using a Bruker maXis II ETD hyphenated with a Shimadzu Nexera system. The instruments were controlled *via* Brukers otofControl 4.1 and Hystar 4.1 SR2 (4.1.31.1) software. The acquisition rate was set to 3 Hz and the following source parameters were used for positive mode electrospray ionization: End plate offset = 500 V; capillary voltage = 3800 V; nebulizer gas pressure = 45 psi; dry gas flow = 10 L/min; dry temperature = 250 °C. Transfer, quadrupole and collision cell settings are mass range dependent and were fine-adjusted with consideration of the respective analyte's molecular weight. For internal calibration sodium format clusters were used. Samples were desalted *via* fast liquid chromatography. A Supelco Titan™ C18 UHPLC Column, 1.9 µm, 80 Å pore size, 20 × 2.1 mm and a 2 min gradient from 10 to 98% aqueous MeCN with 0.1% FA (H_2O: Carl Roth GmbH + Co. KG ROTISOLV® Ultra LC-MS; MeCN: Merck KGaA LiChrosolv® Acetonitrile hypergrade for LC-MS; FA - Merck KGaA LiChropur® Formic acid

98%-100% for LC-MS) was used for separation. Sample dilution in 10% aqueous MeCN (hyper grade) and injection volumes were chosen dependent of the analyte's ionization efficiency. Hence, on-column loadings resulted between 0.25–5.0 ng. Automated internal re-calibration and data analysis of the recorded spectra were performed with Bruker's DataAnalysis 4.4 SR1 software.

UV/Vis spectra were recorded on a Jasco V-770 UV/Vis/NIR Spectrophotometer (PbS-version) with a PAC-743 Peltierthermo 6/8 sample switching unit and a Julabo F-250 cooling system. The spectra were recorded in PBS buffer pH 7.4 using Hellma quartz glass cuvettes (10 mm pathlength).

Fluorescence emission spectra were recorded on a Jasco FP-8600 Fluorescence Spectrometer with PAC-743 Peltierthermo 6/8 sample switching unit and a Julabo F-250 cooling system. The spectra were recorded in PBS buffer pH 7.4 using Hellma dark quartz glass fluorescence cuvettes (10 mm pathlength).

The quantum yields were determined using a Hamamatsu C11347 Absolute PL Quantum Yield Spectrometer (Quantaurus-QY) in PBS pH 7.4.

General protocol to generate NHS esters

A 1 mL vial was charged with 1.0 equiv. of acid dissolved in DMF and 4.0 equiv. of DIPEA was added before 1.1 equiv. of TSTU in one portion (for amounts <1 mg of TSTU, stock solutions were prepared as it is critical to not overload TSTU). The active NHS ester was allowed to form for 15 min and used without further purification.

General protocol for peptide coupling using NHS esters

A 1 mL vial was charged with 1.1 equiv. amine dissolved in DMF and 4.0 equiv. DIPEA. The pre-formed NHS ester (section 1.3) was added drop-wise at and the reaction mixture was allowed to stir at r.t. Upon complete conversion according to LCMS (usually <30 min), the reaction was quenched by addition of 5 vol% HOAc and 10 vol% water and subjected to RP-HPLC. The desired products were obtained as colored powders.

TMR-Mal

TMR-6-COOH: 20.0 mg (46.5 μmol); *N*-(2-Aminoethyl)maleimide trifluoroacetate salt (Aldrich #56951): 13.0 mg (51.1 μmol); 3 mL DMF.

Yield: 11.0 mg; 43%.

Red powder.

^{1}**H NMR** (400 MHz, DMSO-*d*$_6$): δ [ppm] = 8.78 (t, J = 6.0 Hz, 1H), 8.08–8.02 (m, 2H), 7.51 (d, J = 1.3 Hz, 1H), 6.95 (s, 2H), 6.51 (d, J = 1.4 Hz, 6H), 3.52 (t, J = 5.8 Hz, 2H), 3.33 (2H), 2.94 (s, 12H). The signal at 3.33 ppm is colocalized with the water signal at 3.31 ppm, but was detected by COSY and HSQC.

^{13}**C NMR** (100 MHz, DMSO-*d*$_6$): δ [ppm] = 171.0, 168.2, 165.0, 152.8, 152.1, 152.0, 140.5, 134.5, 129.0, 128.6, 128.5, 124.8, 122.3, 109.1, 105.6, 98.0, 84.7, 37.8, 36.9.

UV/Vis (LCMS): λ_{max} = 556 nm.

t_R (LCMS) = 2.502 min.

HRMS (ESI): calc. for $C_{31}H_{29}N_4O_6$ $[M+H]^+$: 553.2082, found: 553.2083.

Cy5-Mal

Cy5-COOH: 100.0 mg (210 µmol); *N*-(2-Aminoethyl)maleimide trifluoroacetate salt (Aldrich #56951): 59.1 mg (232 µmol); 6 mL DMF.

Yield: 35.2 mg; 23%.

Blue powder as its TFA salt.

^{1}H NMR (400 MHz, MeOD-d_4): δ [ppm] = 8.32 (t, J = 13.1 Hz, 2H), 7.89 (t, J = 6.1 Hz, 1H), 7.61 (d, J = 7.4 Hz, 2H), 7.49–7.35 (m, 4H), 7.35–7.13 (m, 2H), 6.99 (s, 2H), 6.56 (t, J = 12.3 Hz, 1H), 6.46–6.16 (m, 2H), 4.07 (t, J = 7.4 Hz, 2H), 3.60 (s, 3H), 3.42 (t, J = 5.8 Hz, 2H), 3.28–3.07 (m, 2H), 1.97 (t, J = 7.2 Hz, 2H), 1.80–1.57 (m, 14H), 1.61–1.43 (m, 2H), 1.40–1.11 (m, 2H).

^{13}C NMR (100 MHz, DMSO-d_6): δ [ppm] = 173.2, 172.5, 172.1, 171.0, 158.1 (q, J = 35.3 Hz), 154.0 (2x), 142.8, 142.0, 141.1, 141.0, 134.5, 128.4, 128.4, 125.3, 124.7, 124.6, 122.4, 122.3, 116.0 (q, J = 293.6 Hz), 111.0 (2x), 103.3, 103.0, 48.9 (2x), 43.3, 37.3, 36.7, 35.0, 31.1, 27.2, 27.0, 26.7, 25.7, 24.7.

UV/Vis (LCMS): λ_{max} = 640 nm.

t_R (LCMS) = 2.971 min.

HRMS (ESI): calc. for $C_{38}H_{45}N_4O_3$ $[M]^+$: 605.3486, found: 605.3488.

SiR-Mal

SiR-6-COOH: 30.0 mg (63.4 μmol); *N*-(2-Aminoethyl)maleimide trifluoroacetate salt (Aldrich #56951): 17.7 mg (69.6 μmol); 4 mL DMF.

Yield: 22.8 mg; 60%.

Blue powder.

^{1}H NMR (400 MHz, DMSO-d_6): δ [ppm] = 8.82 (t, J = 6.0 Hz, 1H), 8.02 (d, J = 8.0 Hz, 1H), 7.94 (dd, J = 8.0, 1.4 Hz, 1H), 7.62–7.57 (m, 1H), 7.02 (t, J = 2.7 Hz, 2H), 6.96 (s, 2H), 6.72–6.55 (m, 4H), 3.54 (dd, J = 6.5, 5.0 Hz, 2H), 2.92 (d, J = 1.4 Hz, 12H), 0.64 (s, 3H), 0.53 (s, 3H). The signal at 3.33 ppm is colocalized with the water signal at 3.31 ppm, but was detected by COSY.

^{13}C NMR (100 MHz, DMSO-d_6): δ [ppm] = 171.0, 169.3, 165.3, 154.7, 149.3, 139.9, 135.9, 135.3, 134.5, 130.4, 128.0, 127.5, 127.4, 125.4, 122.7, 116.4, 113.7, 91.3, 37.8, 36.9, 0.1, -1.2.

UV/Vis (LCMS): λ_{max} = 656 nm.

$\boldsymbol{t_R}$ (LCMS) = 2.514 min.

HRMS (ESI): calc. for $C_{33}H_{35}N_4O_5Si$ $[M+H]^+$: 595.2371, found: 595.2374.

Synthesis of S39C-Ex4(9-39)

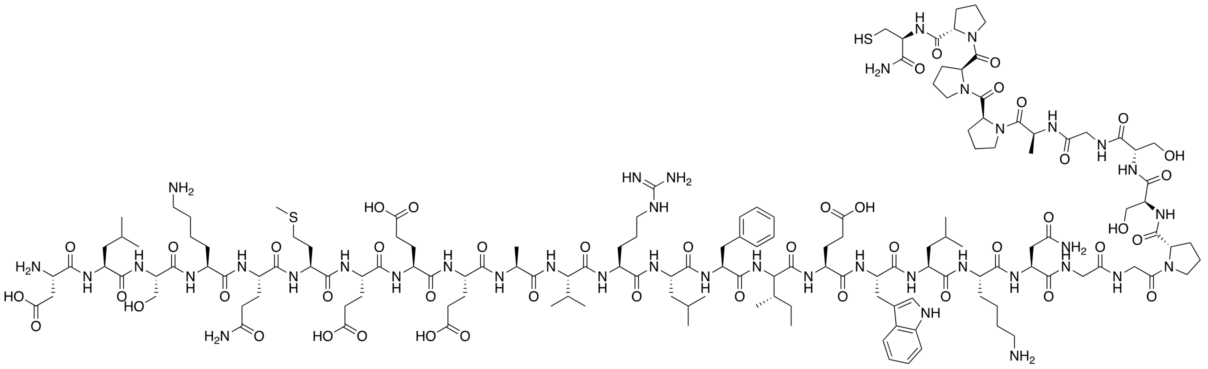

S39C-Ex4(9-39)

H-Asp-Leu-Ser-Lys-Gln-Met-Glu-Glu-Glu-Ala-Val-Arg-Leu-Phe-Ile-Glu-Trp-Leu-Lys-Asn-Gly-Gly-Pro-Ser-Ser-Gly-Ala-Pro-Pro-Pro-Cys-NH_2

Chemical Formula: $C_{149}H_{234}N_{40}O_{46}S_2$
Exact Mass: 3383,6642
Molecular Weight: 3385,8650

Peptides were synthesized on a CEM Liberty Blue Peptide Synthesizer with a CEM Discovery Microwave using standard Fmoc-protected solid phase peptide synthesis protocols with standard reagents. Pre-loaded Fmoc-Cys(Trt)-Tentagel S PHB resin (Rapp Polymere, Germany) containing 0.2-0.3 mmol/g amino acid was used as solid-phase. Peptide synthesis scale was 0.1 mmol using the standard coupling reagents DIC/Oxyma 0.5/1.0 M in DMF and DIPEA 2 M in DMF. Fmoc-protected amino acids (Sigma-Aldrich and NovaBiochem Merck, Germany) with standard residual protecting groups were coupled using a five-fold excess (2 M solutions). Deprotection of the Fmoc-protecting group was achieved by treatment with 20% piperidine in DMF. After completion of all coupling steps, the resin-bound peptide was transferred into a syringe with frit followed by global deprotection using 10 mL of a TFA:H_2O:tri-*iso*-propylsilane (95:2.5:2.5) mixture within 2 h under argon atmosphere. The peptide solution was filtered and the filtrate was concentrated under reduced pressure and residual TFA was removed by co-evaporation with toluene (3x). The residue was dissolved in a small amount of methanol and precipitated in 40 mL chilled diethyl ether and stored overnight at –38 °C to complete precipitation. The suspension was subjected to centrifugation (1843 x g, 4 °C, 5 min), the supernatant removed and the residue dried before being reconstituted in water and subjected to RP-HPLC purification. The combined purified fractions were lyophilized and the **S39C-Ex4(9-39)** peptide was yielded as white TFA salt.

Yield: 50 mg; ~15%.

UV/Vis (LCMS): λ_{max} = 212 nm.

t_R (HPLC) = 24.4 min.

HRMS (ESI): calc. for $C_{149}H_{238}N_{40}O_{46}S_2$ $[M+4H]^{4+}$: 847.1742, found: 847.1741.

Synthesis of LUXendin555

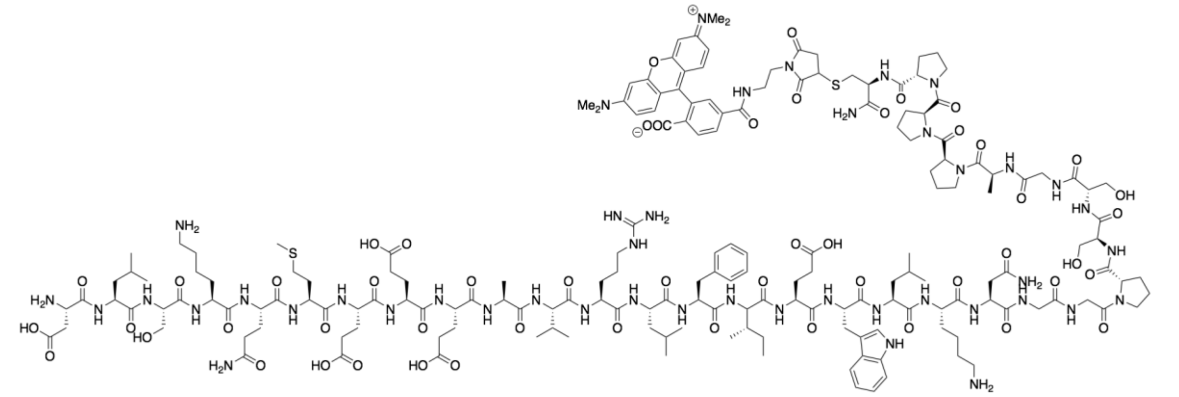

LUXendin555

H-Asp-Leu-Ser-Lys-Gln-Met-Glu-Glu-Glu-Ala-Val-Arg-Leu-Phe-Ile-Glu-Trp-Leu-Lys-Asn-Gly-Gly-Pro-Ser-Ser-Gly-Ala-Pro-Pro-Pro-Cys(TMR)-NH_2

Chemical Formula: $C_{180}H_{262}N_{44}O_{52}S_2$
Exact Mass: 3935,8651
Molecular Weight: 3938,4520

To a solution of S39C-Ex4(9-39) (2.03 mg, 600 nmol, 1.0 eq.) in PBS (400 μL) was added TMR-Mal (0.5 mg, 877 nmol, 1.5 eq.). The solution was stirred at room temperature over night before being subjected to RP-HPLC purification (water/ACN gradient, 90/10 → 10/90 in 60 min). The purified fractions were combined and lyophilized from the HPLC solvent to yield **LUXendin555** as light blue TFA salt. The concentration was determined *via* the absorption of the TMR fluorophore at 550 nm (ε = 84,000 mol L^{-1} cm^{-1} in PBS with 0.1% SDS) and stocks of 10 nmol each were prepared and stored at -80 °C.

Yield: 350 nmol; 58%.
UV/Vis (UV, FluoSpec): Ex_{max} = 555 nm, Em_{max} = 579 nm.
t_R (HPLC) = 25.0 min.
HRMS (ESI): calc. for $C_{180}H_{266}N_{44}O_{52}S_2$ $[M+4H]^{4+}$: 985.4764, found: 985.4749.

Synthesis of LUXendin645

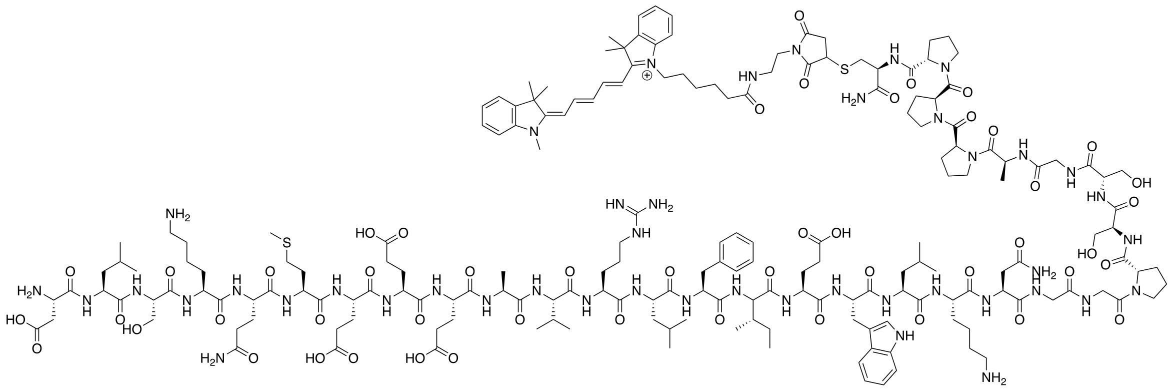

LUXendin645

H-Asp-Leu-Ser-Lys-Gln-Met-Glu-Glu-Glu-Ala-Val-Arg-Leu-Phe-Ile-Glu-Trp-Leu-Lys-Asn-Gly-Gly-Pro-Ser-Ser-Gly-Ala-Pro-Pro-Pro-Cys(Cy5)-NH_2

Chemical Formula: $C_{187}H_{279}N_{44}O_{49}S_2^+$
Exact Mass: 3989,0128
Molecular Weight: 3991,6675

To a solution of S39C-Ex4(9-39) (2.03 mg, 600 nmol, 1.0 eq.) in PBS (400 μL) was added Cy5-Mal (0.5 mg, 824 nmol, 1.4 eq.). The solution was stirred at room temperature over night before being subjected to RP-HPLC purification (water/ACN gradient, 90/10 → 10/90 in 60 min). The purified fractions were combined and lyophilized from the HPLC solvent to yield **LUXendin645** as light blue TFA salt. The concentration was determined *via* the absorption of the Cy5 fluorophore at 647 nm (ε = 250,000 mol L^{-1} cm^{-1} in PBS with 0.1% SDS) and stocks of 10 nmol each were prepared and stored at -80 °C.

Yield: 400 nmol; 67%.
UV/Vis (UV, FluoSpec): Ex_{max} = 645 nm, Em_{max} = 664 nm.
$\boldsymbol{t_R}$ (HPLC) = 26.3 min.
HRMS (ESI): calc. for $C_{187}H_{279}N_{44}O_{49}S_2$ $[M+4H]^{5+}$: 799.0097, found: 799.0095.

Synthesis of LUXendin651

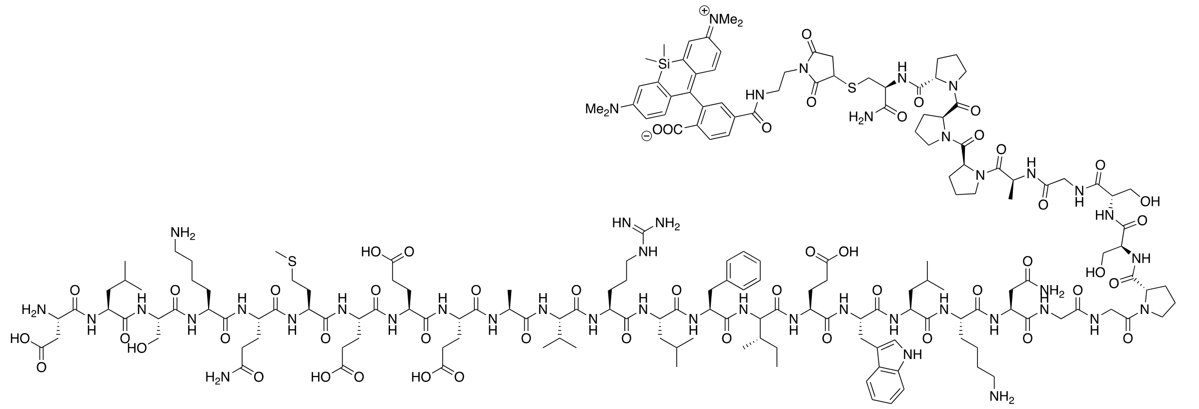

LUXendin651

H-Asp-Leu-Ser-Lys-Gln-Met-Glu-Glu-Glu-Ala-Val-Arg-Leu-Phe-Ile-Glu-Trp-Leu-Lys-Asn-Gly-Gly-Pro-Ser-Ser-Gly-Ala-Pro-Pro-Pro-Cys(SiR)-NH_2

Chemical Formula: $C_{182}H_{268}N_{44}O_{51}S_2Si$
Exact Mass: 3977,8941
Molecular Weight: 3980,6080

To a solution of S39C-Ex4(9-39) (2.03 mg, 600 nmol, 1.0 eq.) in PBS (400 μL) was added SiR-Mal (0.5 mg, 833 nmol, 1.4 eq.). The solution was stirred at room temperature over night before being subjected to RP-HPLC purification (water/ACN gradient, 90/10 → 10/90 in 60 min). The purified fractions were combined and lyophilized from the HPLC solvent to yield **LUXendin651** as light blue TFA salt. The concentration was determined *via* the absorption of the SiR fluorophore at 647 nm (ε = 100,000 mol L^{-1} cm^{-1} in PBS with 0.1% SDS) and stocks of 10 nmol each were prepared and stored at -80 °C.

Yield: 400 nmol; 67%.

UV/Vis (UV, FluoSpec): Ex_{max} = 651 nm, Em_{max} = 669 nm.

$\boldsymbol{t_R}$ (HPLC) = 24.8 min.

HRMS (ESI): calc. for $C_{182}H_{269}N_{44}O_{51}S_2Si$ $[M+4H]^{4+}$: 995.9836, found: 995.9821.

SUPPLEMENTARY FIGURES

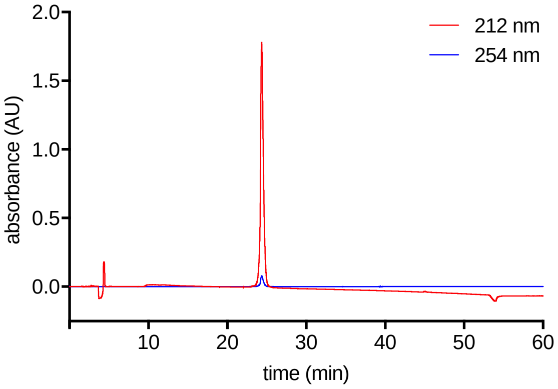

Supplementary Figure 1: Analytical RP-HPLC trace of S39C-Ex4(9-39) peptide

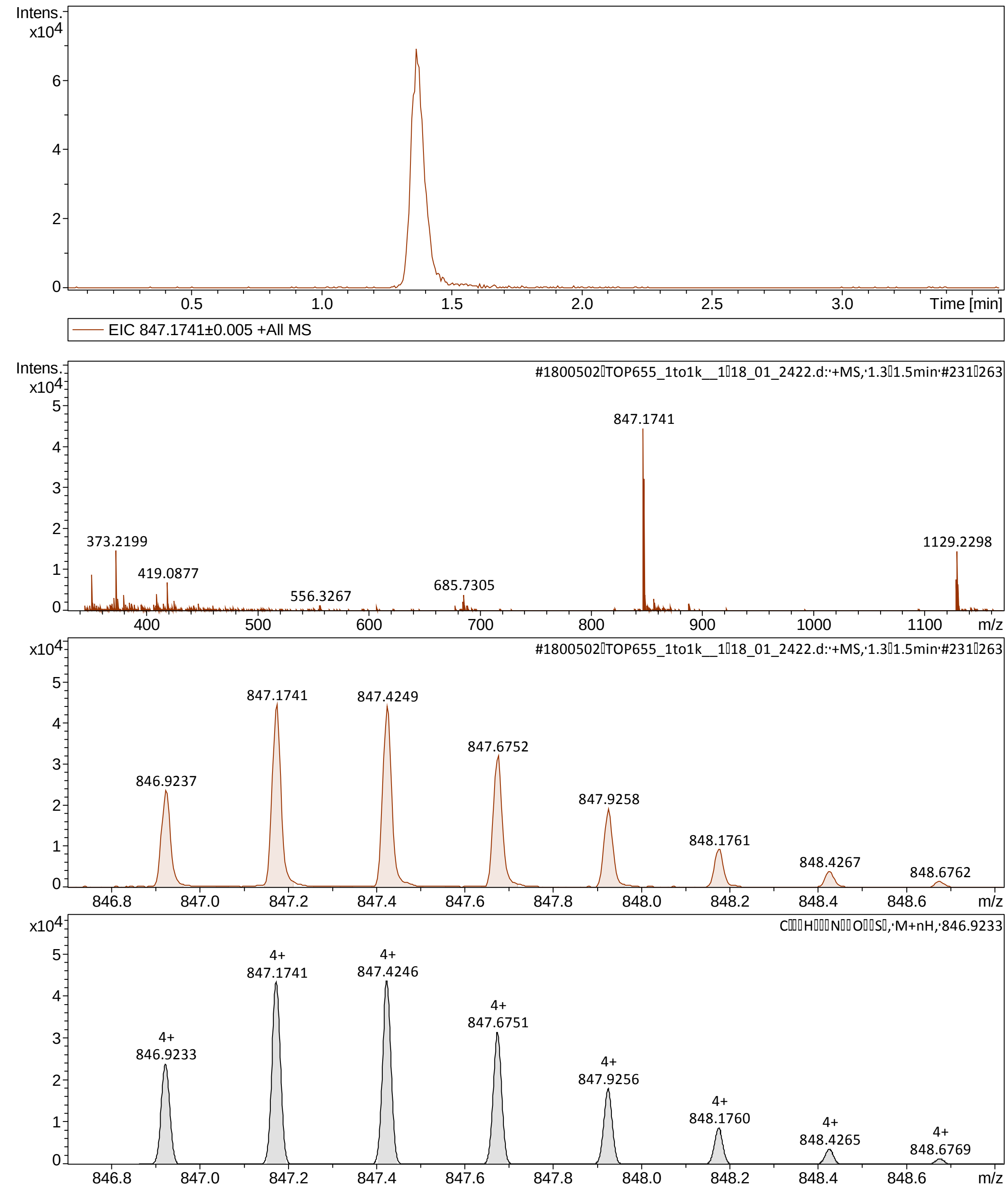

Supplementary Figure 2: HR ESI-MS spectrum of S39C-Ex4(9-39).

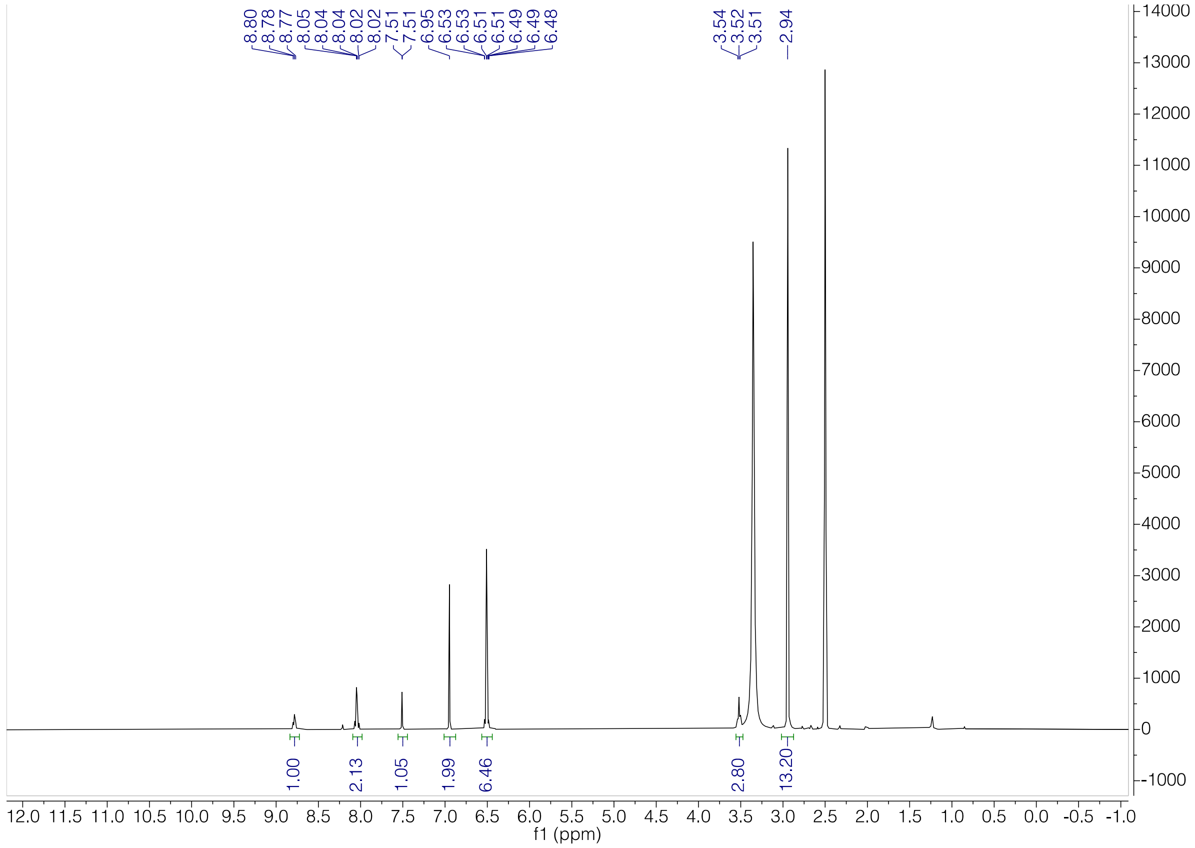
8.80
8.78
8.77
8.05
8.04
8.02
8.02
7.51
7.51
6.95
6.53
6.53
6.51
6.51
6.49
6.48
3.54
3.52
3.51
2.94
1.00
2.13
1.05
1.99
6.46
2.80
13.20
f1 (ppm)

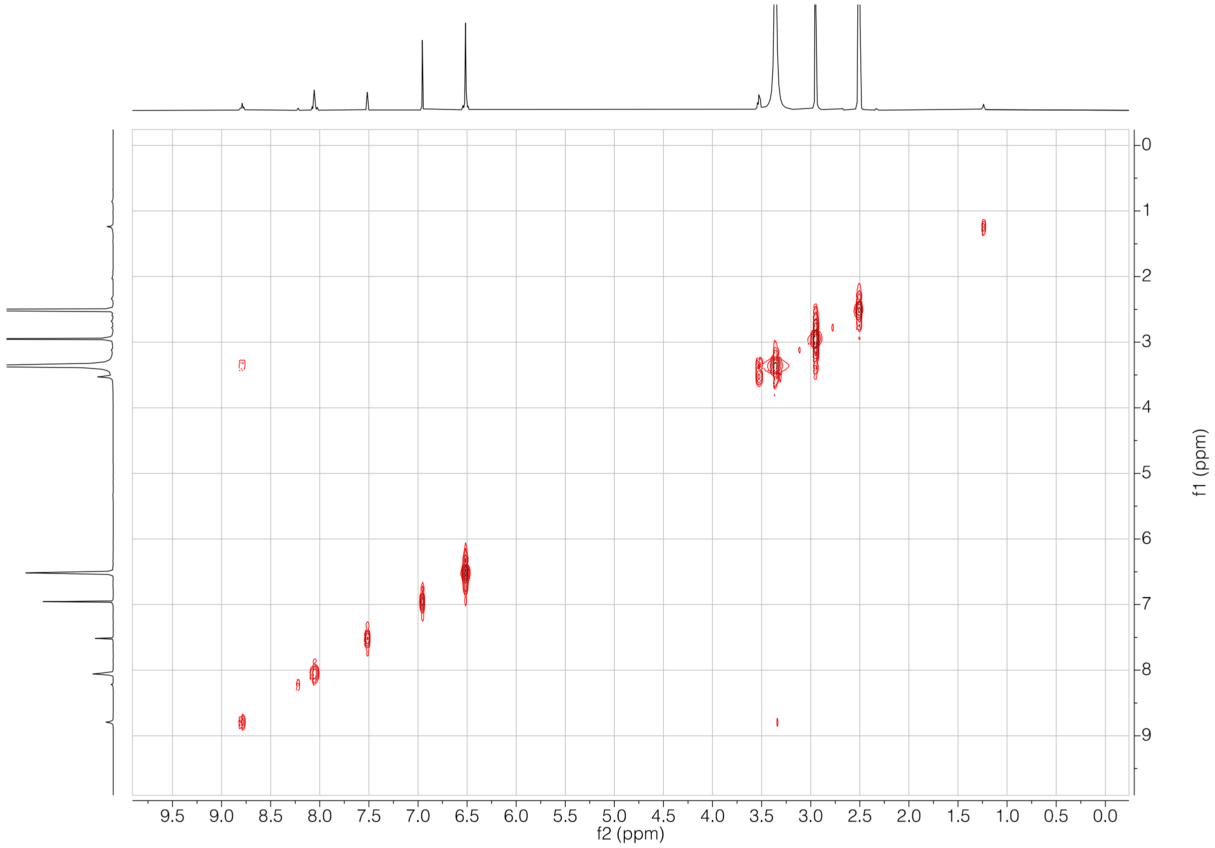
f2 (ppm)
f1 (ppm)

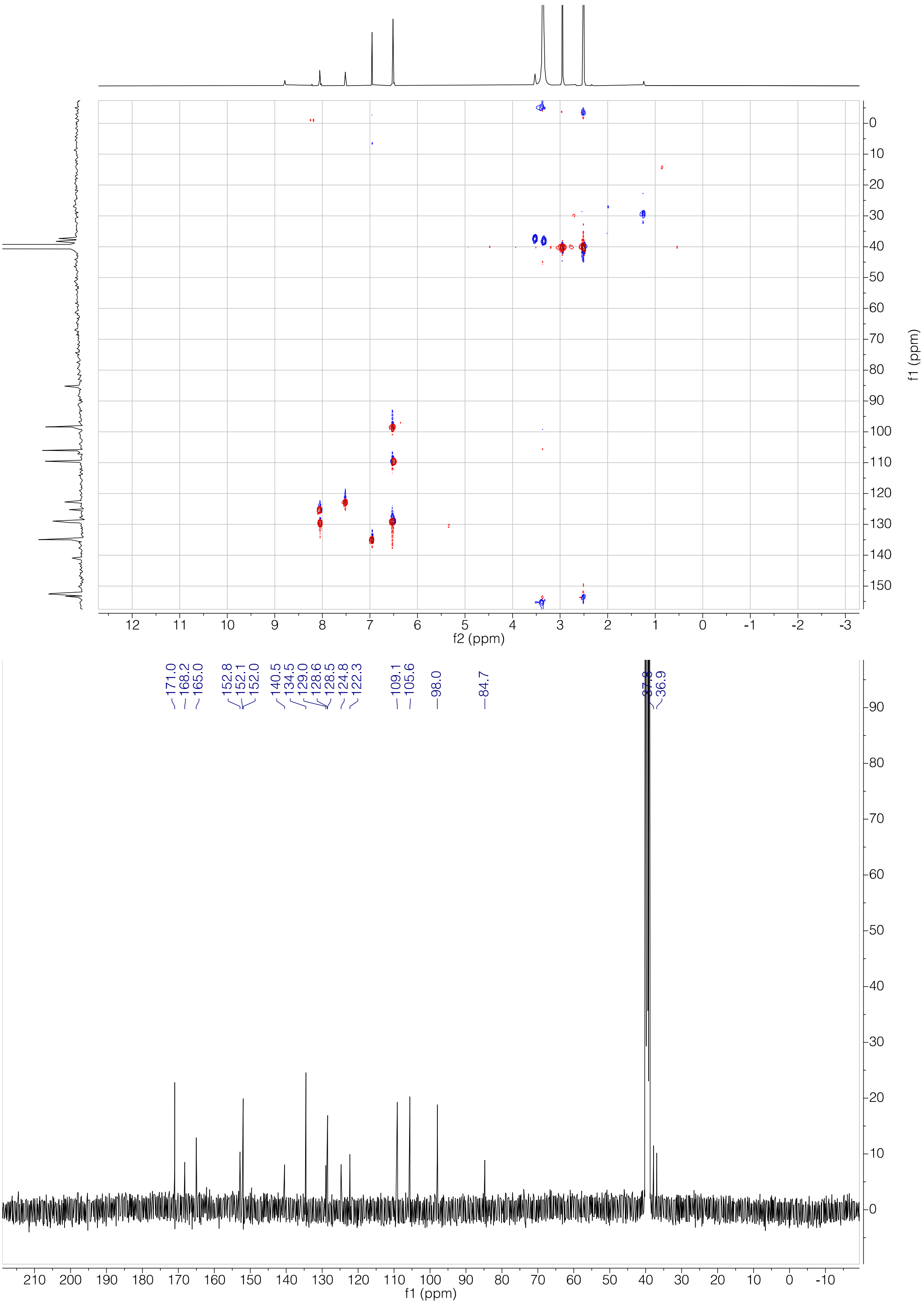

Supplementary Figure 3: NMR spectroscopy of TMR-Mal: ^{1}H, COSY, HSQC and ^{13}C.

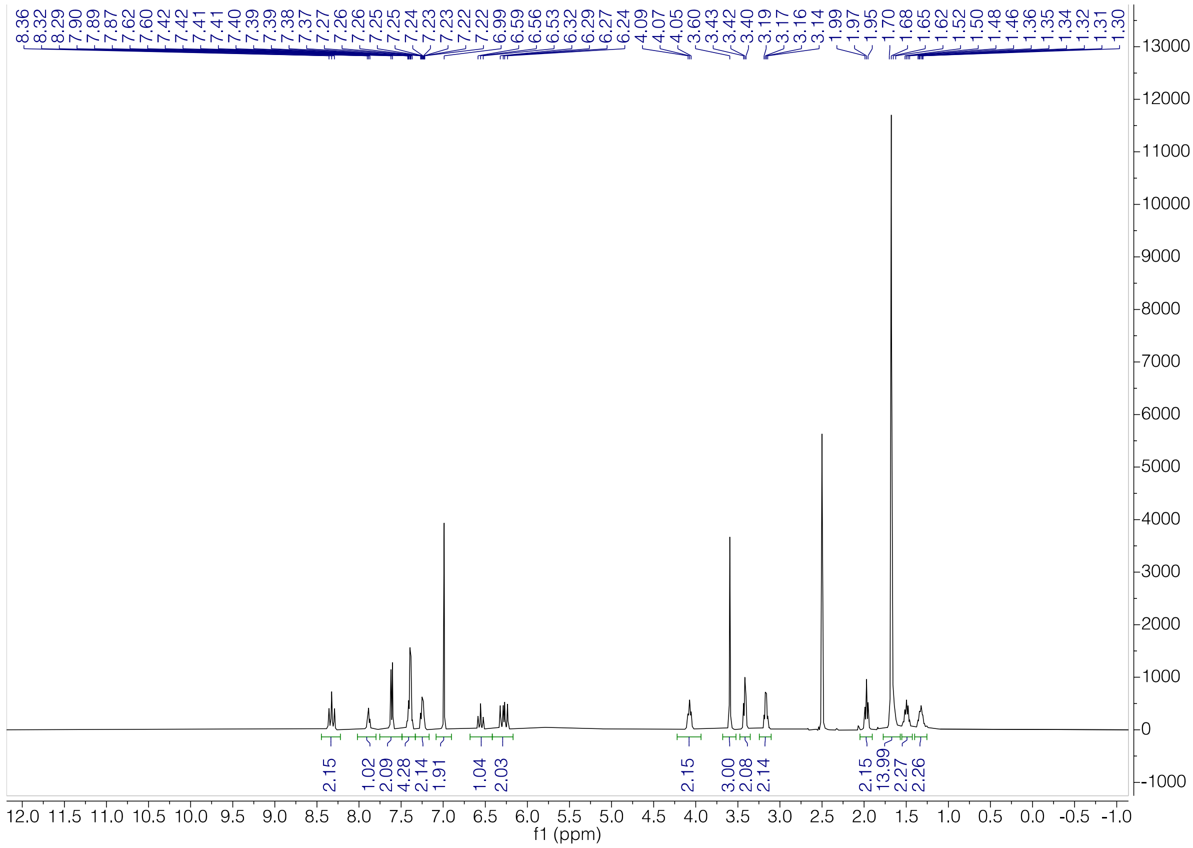
8.36 8.32 8.29 7.90 7.89 7.87 7.62 7.60 7.42 7.42 7.41 7.40 7.39 7.39 7.38 7.37 7.27 7.26 7.25 7.25 7.24 7.23 7.23 7.22 7.22 6.99 6.59 6.56 6.53 6.32 6.29 6.27 6.24 4.09 4.07 4.05 3.60 3.43 3.42 3.40 3.19 3.17 3.16 3.14 1.99 1.97 1.95 1.70 1.68 1.65 1.62 1.52 1.50 1.48 1.46 1.36 1.35 1.34 1.32 1.31 1.30
2.15 1.02 2.09 4.28 2.14 1.91 1.04 2.03 2.15 3.00 2.08 2.14 2.15 13.99 2.27 2.26
f1 (ppm)

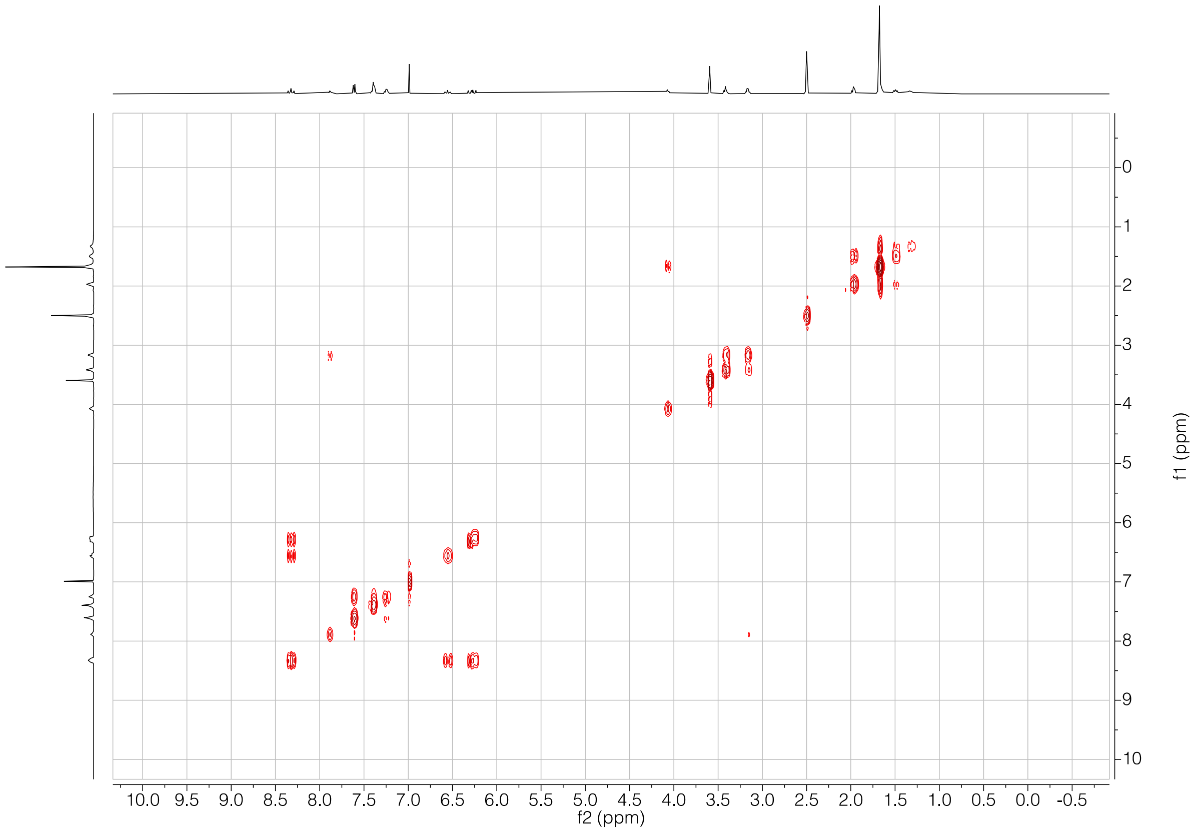
f2 (ppm)
f1 (ppm)

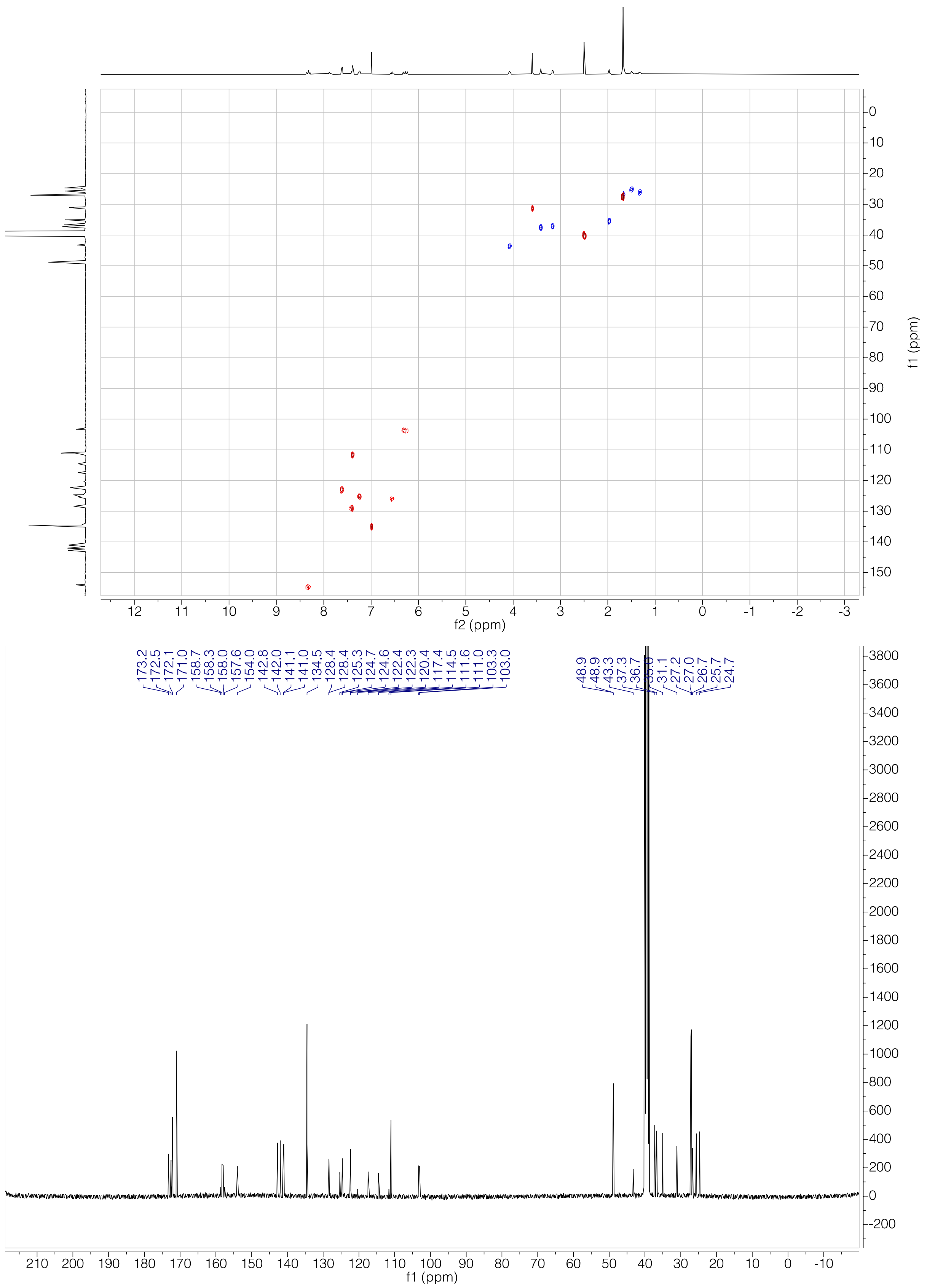

Supplementary Figure 4: NMR spectroscopy of Cy5-Mal: ^{1}H, COSY, HSQC and ^{13}C.

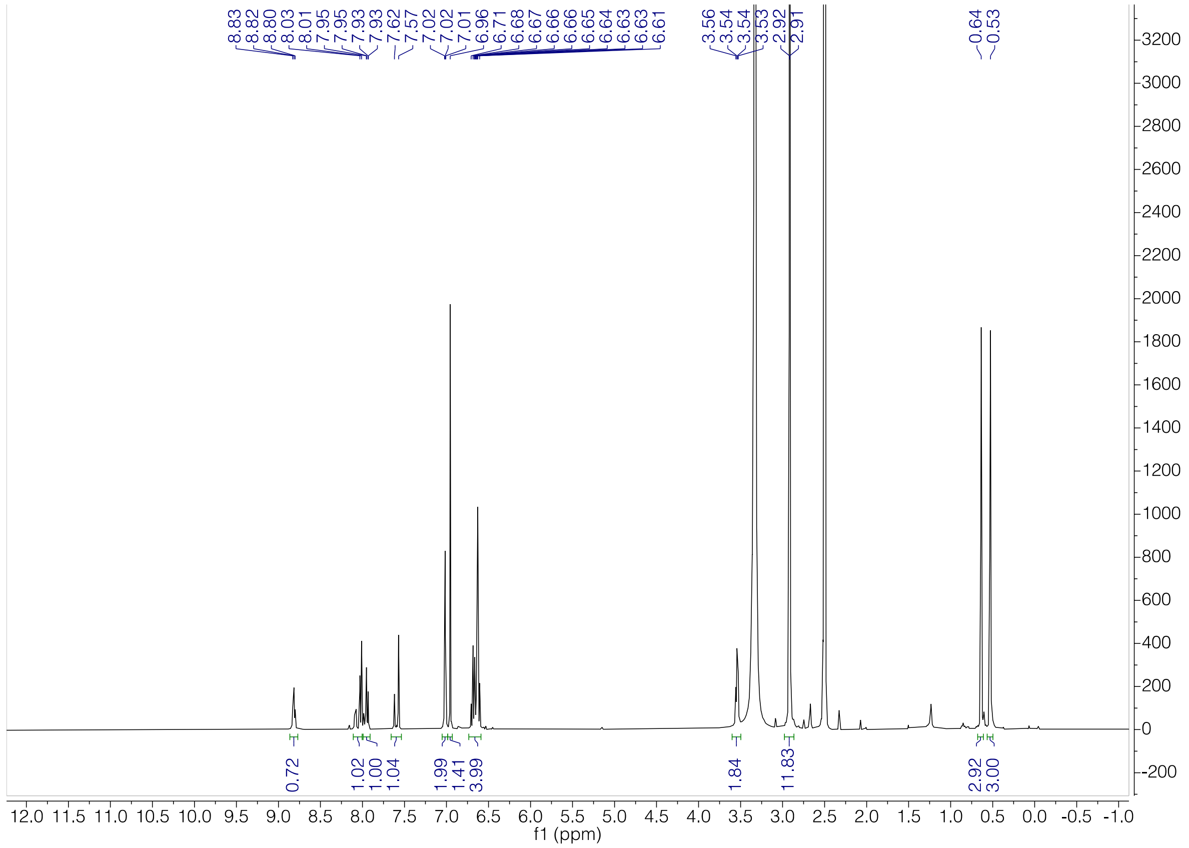
8.83
8.82
8.80
8.03
8.01
7.95
7.95
7.93
7.93
7.62
7.57
7.02
7.02
7.01
6.96
6.71
6.68
6.67
6.66
6.66
6.65
6.64
6.63
6.63
6.61
3.56
3.54
3.54
3.53
2.92
2.91
0.64
0.53
0.72
1.02
1.00
1.04
1.99
1.41
3.99
1.84
11.83
2.92
3.00
f1 (ppm)

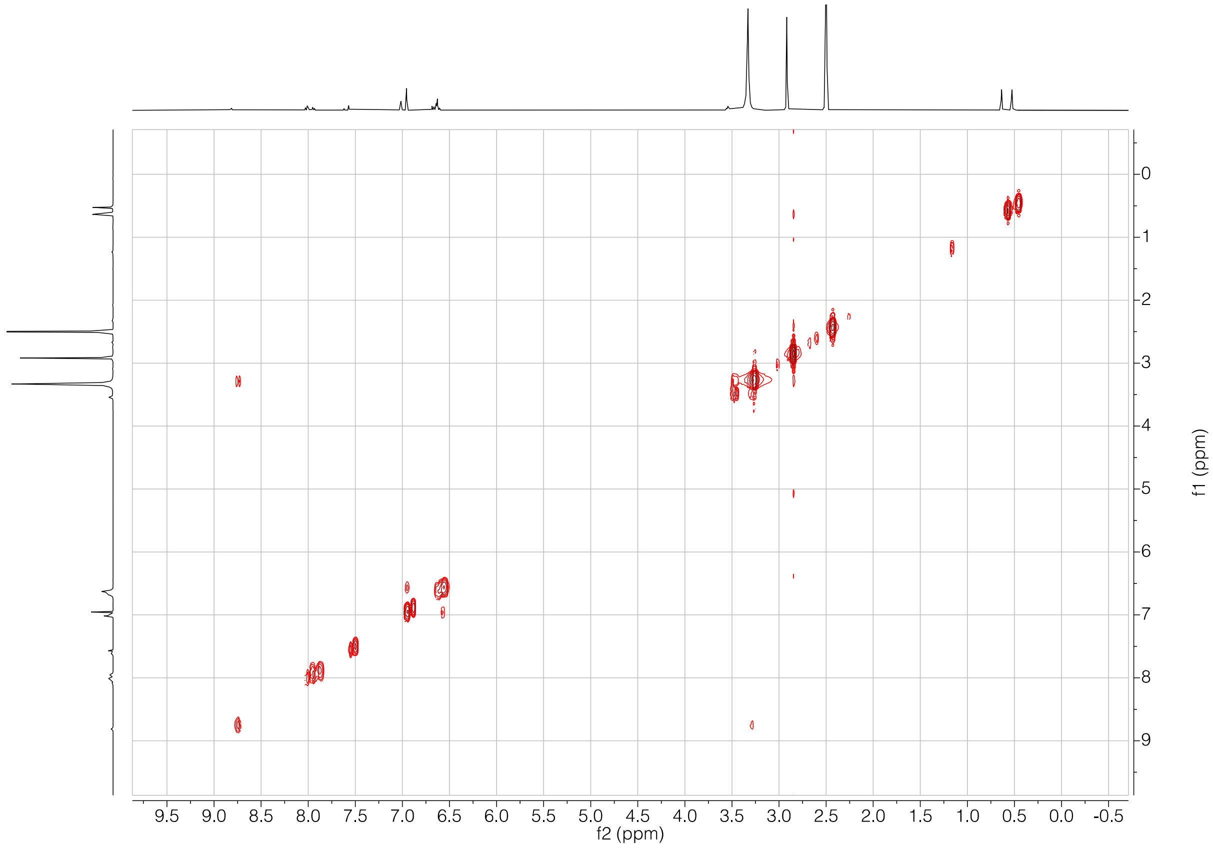
f2 (ppm)
f1 (ppm)

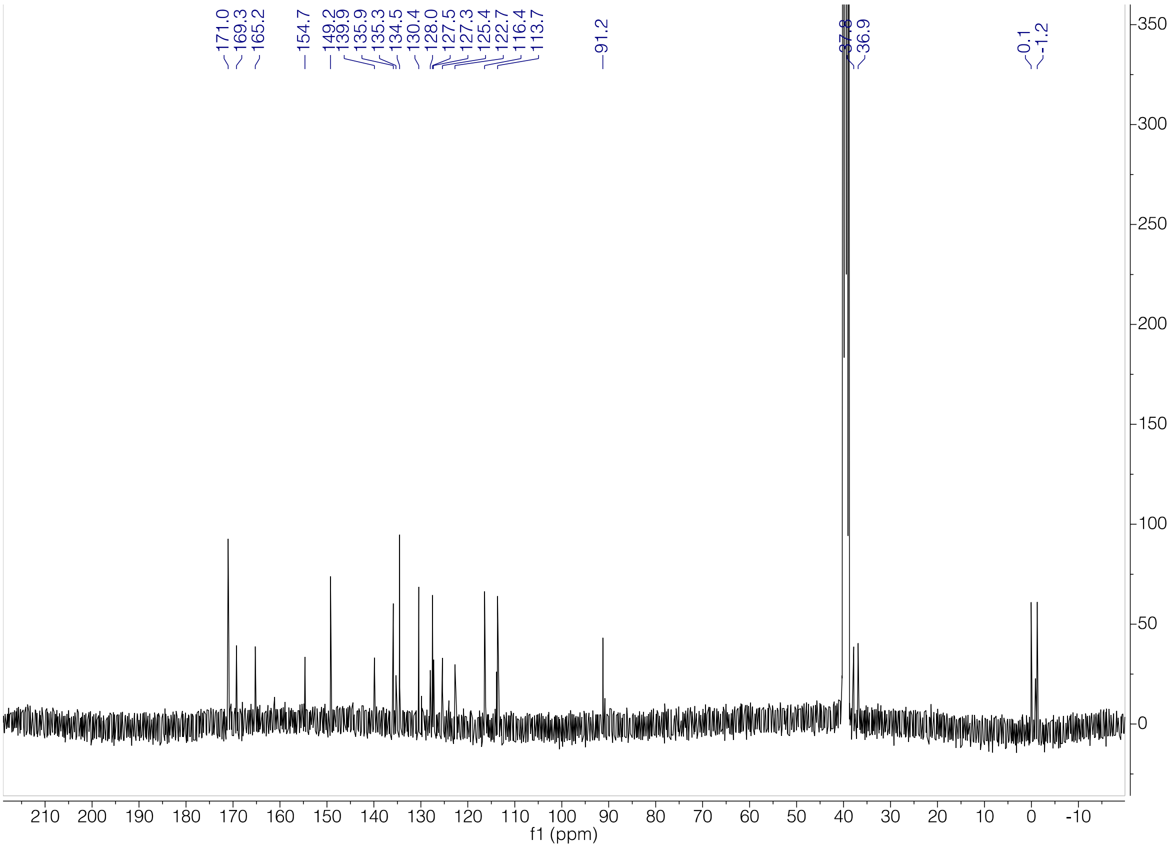

Supplementary Figure 5: NMR spectroscopy of SiR-Mal: ^{1}H, COSY and ^{13}C.

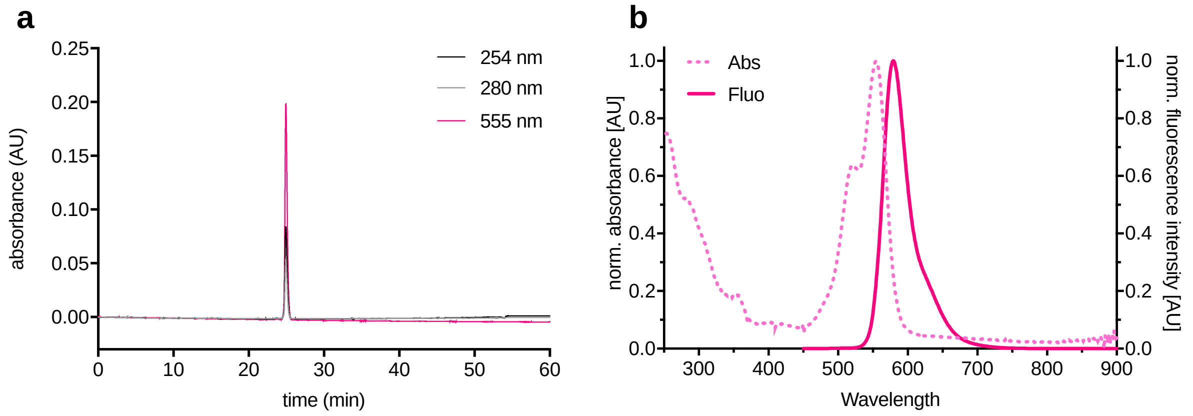

Supplementary Figure 6: LUXendin555 HPLC and absorbance/emission spectra. a Analytical RP-HPLC trace of **LUXendin555**. **b** Normalized absorption and fluorescence emission spectra of **LUXendin555**.

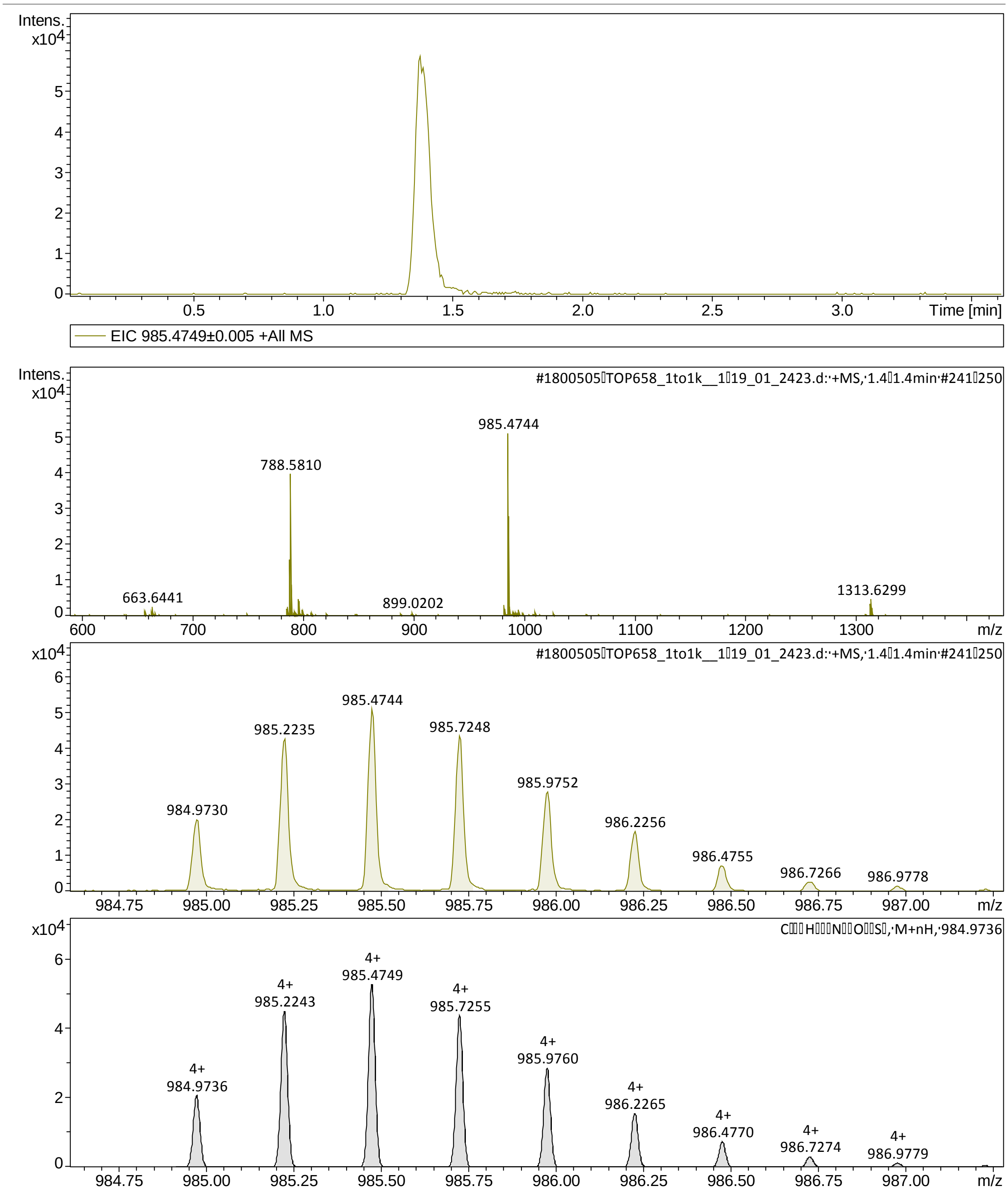

Supplementary Figure 7: HR ESI-MS of LUXendin555.

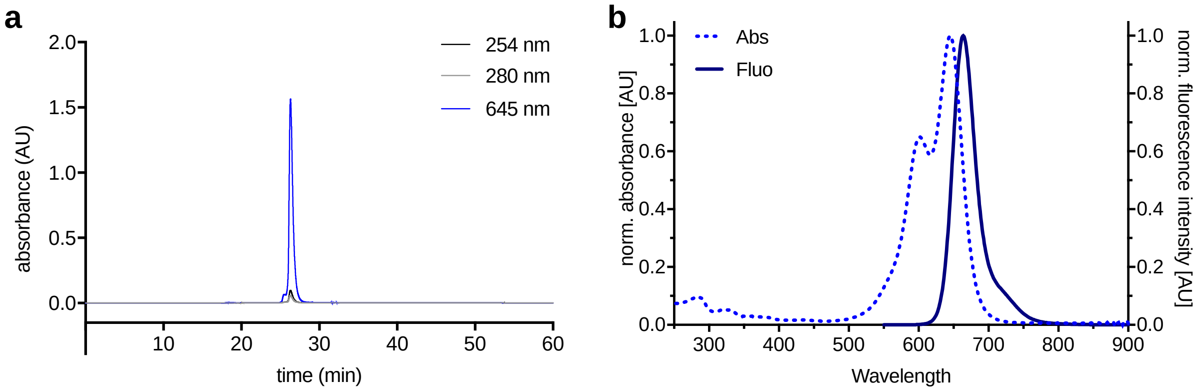

Supplementary Figure 8: LUXendin645 HPLC and absorbance/emission spectra. a Analytical RP-HPLC trace of **LUXendin645**. **b** Normalized absorption and fluorescence emission spectra of **LUXendin645**.

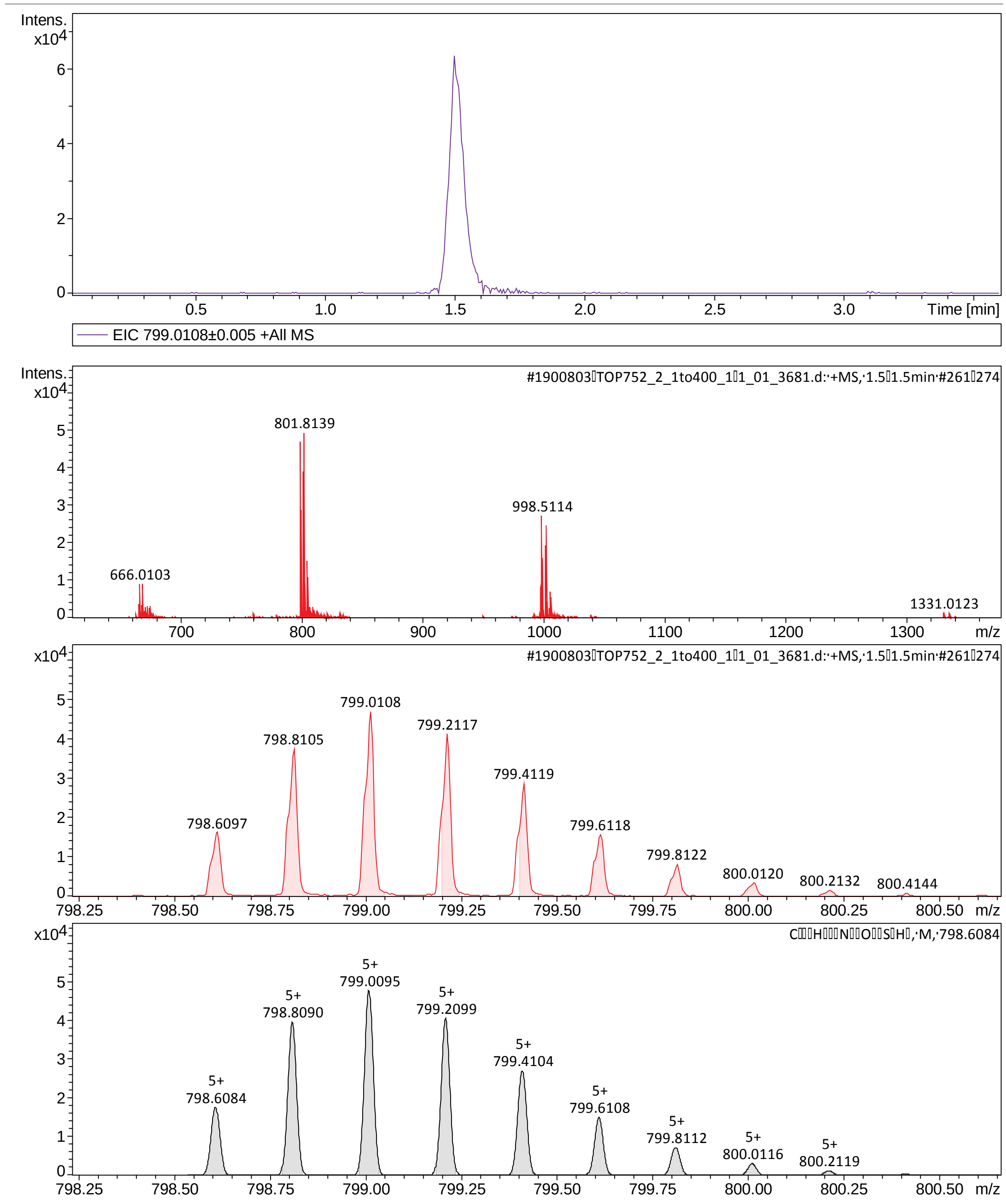

Supplementary Figure 9: HR ESI-MS of LUXendin645.

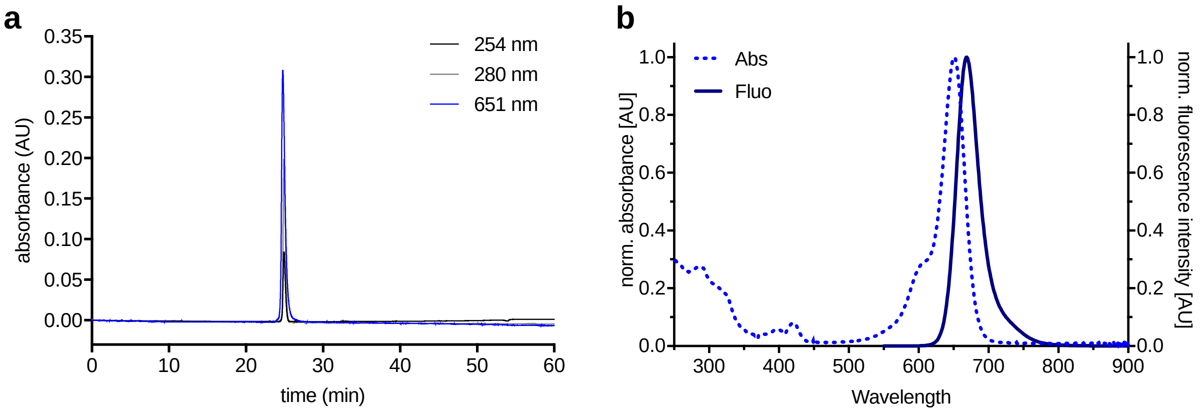

Supplementary Figure 10: LUXendin651 HPLC and absorbance/emission spectra. a Analytical RP-HPLC trace of **LUXendin651**. **b** Normalized absorption and fluorescence emission spectra of **LUXendin651**.

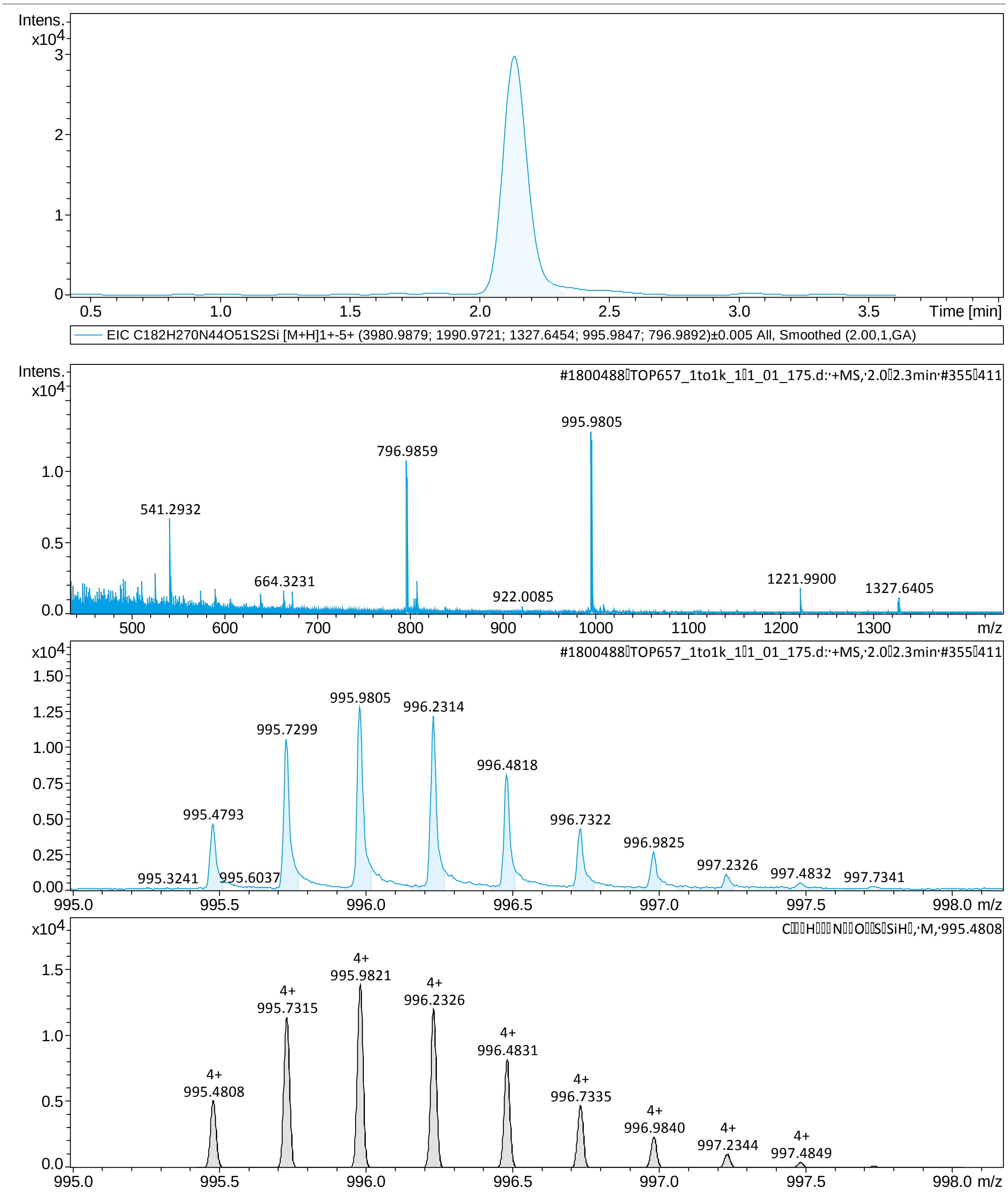

Supplementary Figure 11. HR ESI-MS of LUXendin651.

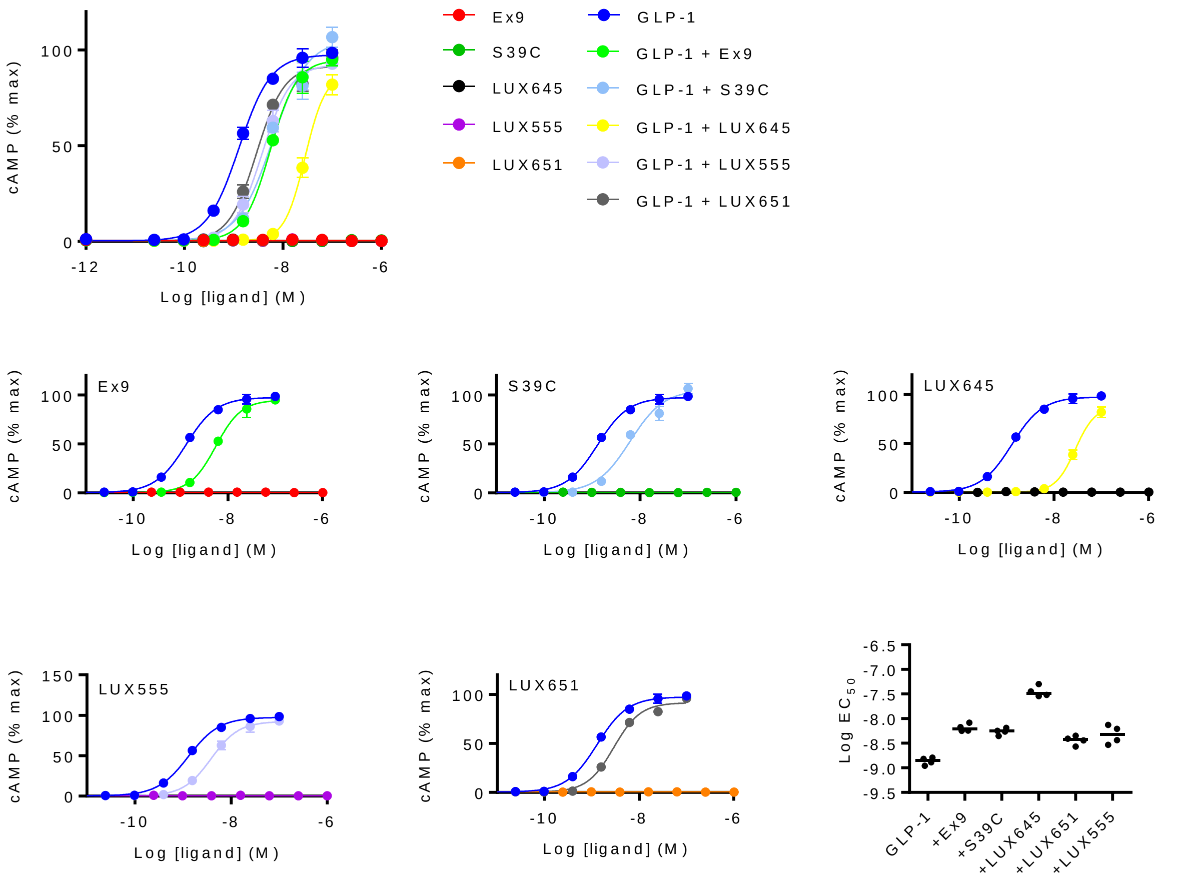

Supplementary Figure 12: LUXendin645, LUXendin651 and LUXendin555 cAMP pharmacology. HTRF cAMP assays in HEK-SNAP_GLP1R cells in agonist and antagonist modes, stimulated with ligand for 30 min. Note that **LUXendins** are GLP1R antagonists (applied at 1 µM), with little to no agonist activity (n = 4 independent assays). Four-parameter fits are displayed, with LogEC_{50} estimates shown as individual replicates. GLP-1, glucagon-like peptide-1; Ex9, Exendin4(9-39); S39C, S39C_Exendin4(9-39). Mean ± SEM are shown. Source data are provided as a Source Data file.

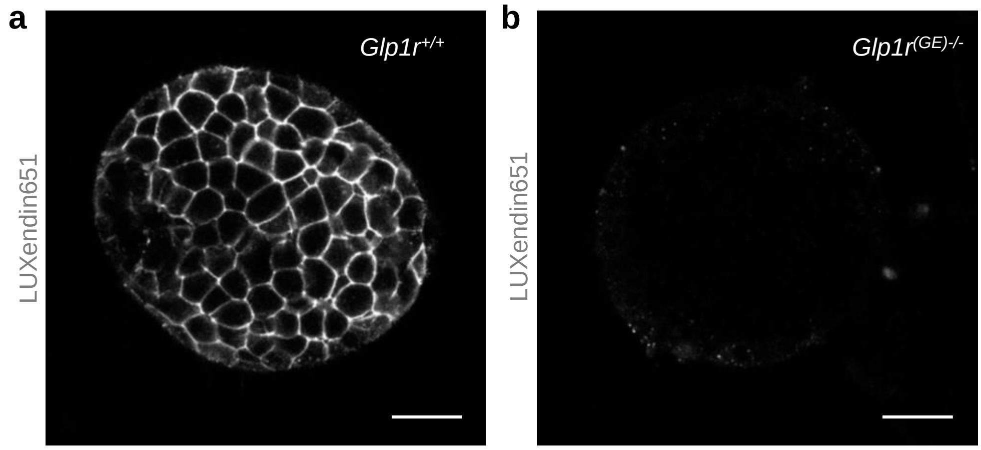

Supplementary Figure 13: LUXendin651 specifically labels GLP1R. a, b Signal can be detected in wild-type (**a**) but not *Glp1r*$^{(GE)-/-}$ islets (**b**) (n = at least 30 islets for each genotype, at least 6 animals per genotype, 3 preparations) (scale bar = 26.5 µm). **LUXendin651** was applied at 100 nM.

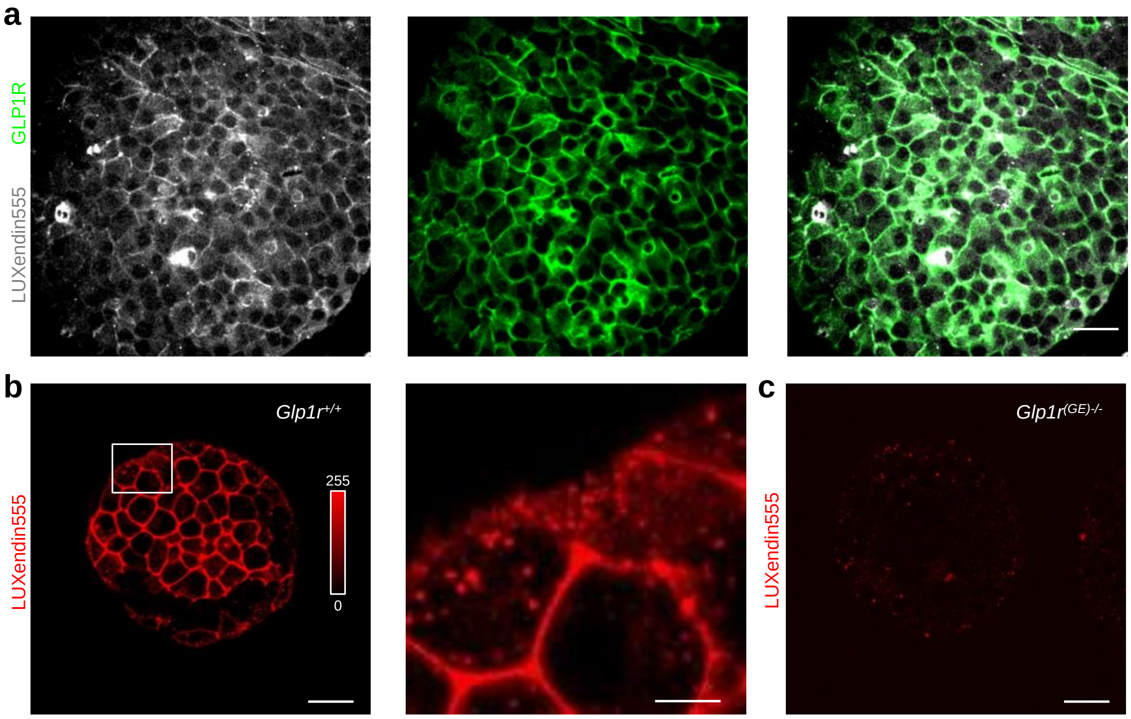

Supplementary Figure 14: LUXendin555 specifically labels GLP1R. a Some punctate staining is seen with 250 nM **LUXendin555** labeling, but not in the same samples co-stained with monoclonal antibody against GLP1R. **b**, **c** Signal can be detected in wild-type (**b**) but not *Glp1r*$^{(GE)-/-}$ islets (**c**) labelled with 250 nM **LUXendin555** (inset shows punctate staining) (n = at least 33 islets for each genotype, at least 6 animals per genotype, 3 preparations) (scale bar = 26.5 µm for full-field images, 6.5 µm for zoomed-in image). Note that the brightness/contrast in image (**c**) has been increased relative to image (**b**) to allow the *Glp1r*$^{(GE)-/-}$ islet to be seen. The white box shows the location of the zoom-in.

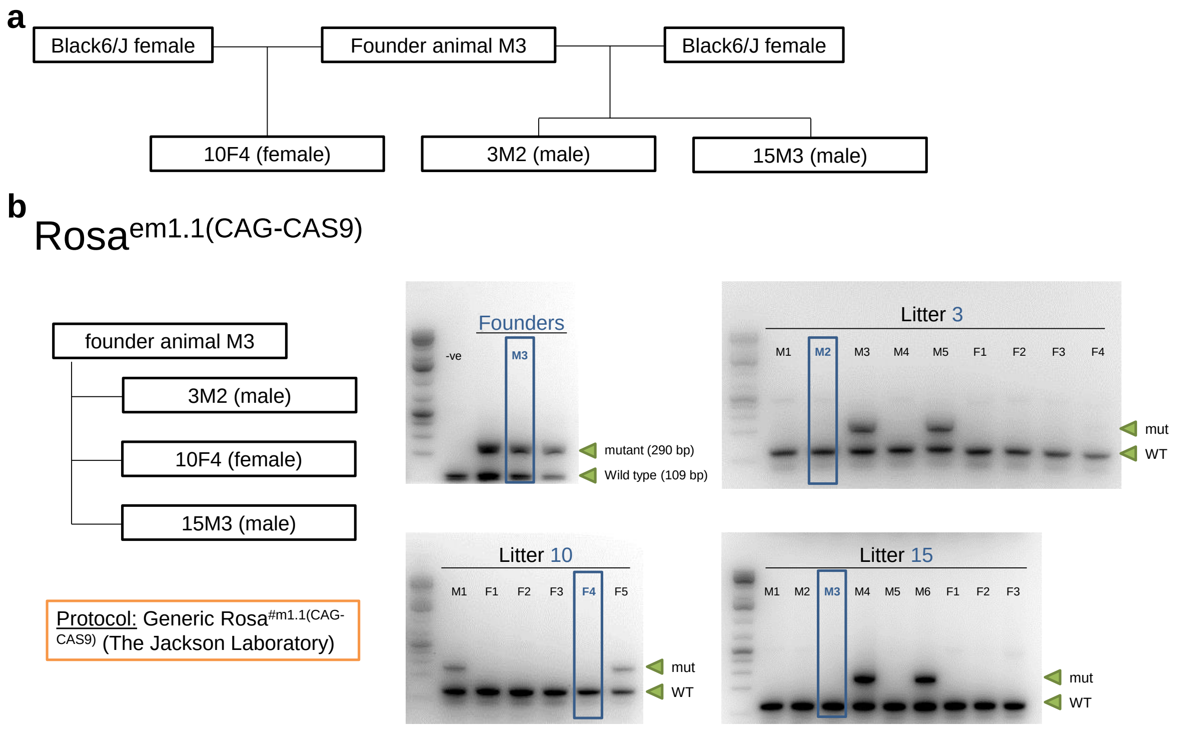

Supplementary Figure 15: Breeding strategy for *Glp1r*$^{(GE)-/-}$ mice. a The founder animal carrying a mutant *Glp1r* allele was paired with two wild type (WT) Black6/J females. Three of their offspring (10F4, 3M2, 15M3) carrying the knock-out *Glp1r* allele were used for further breeding. **b** Founder animal M3 was heterozygous for Rosa$^{em1.1(CAG\text{-}CAS9)}$, as shown by PCR following The Jackson Laboratory protocol 'Generic Rosa$^{\#m1.1(CAG\text{-}CAS9)}$'. None of the three offspring used for further breeding expressed the Cas9 protein. Full, uncropped gels are provided in the Source Data file.

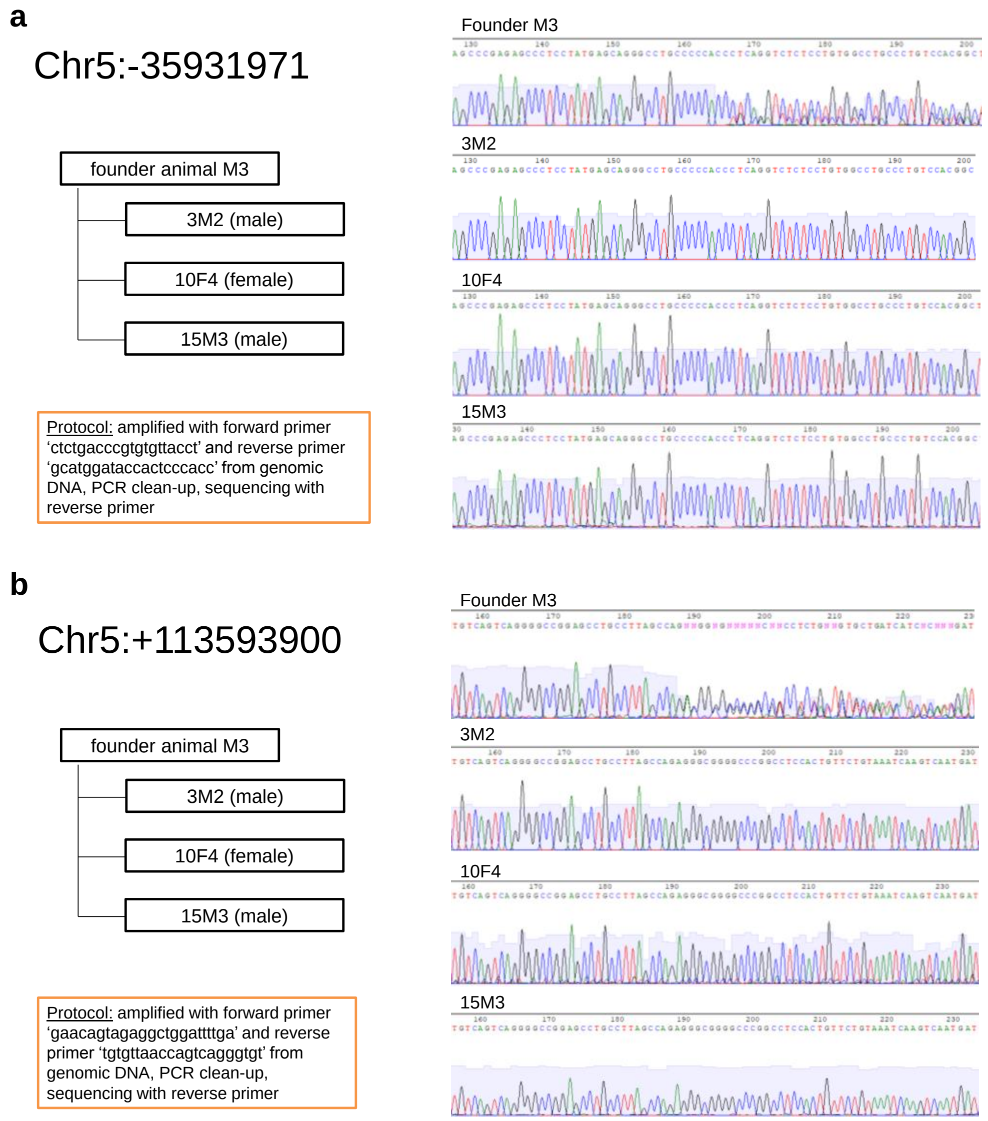

Supplementary Figure 16: Screening *Glp1r*$^{(GE)-/-}$ mice for off-target mutations. a Amplification of chr5:-35931971 and sequencing of the resulting PCR product revealed changes after CRISPR/Cas9 genome editing in the founder animal M3. Offspring 3M2, 10F4 and 15M3 were analyzed in the same way and showed no off-target effects. **b** As for a, but amplification and screening of chr5:+113593900 showing a mutation after CRISPR/Cas9 genome editing in the founder animal but not in the next generation. Source data are provided as a Source Data file.

Locus	forward primer	reverse primer	Affected in Founder M3?
chr9:-114887514	GACTGCAAGGTTCGAGGAGC	CGGGAAATGCCTGACTCATAC	no
chr5:-35931971	CTCTGACCCGTGTGTTACCT	GCATGGATACCACTCCCACC	yes
chr1:-138015670	TGGAACAGAGCGGCATACTC	AATAAGGGTCGTTTGGGAGCC	no
chr4:+71861176	TAGACATCCAGTGGGAGGGG	GAGCCGCCCGCAATGT	no
chr4:+53051322	AAGCGTTTTGATTTTTGTCTGGC	TGACGGGAAGAGAGCATGTG	no
chr9:-53418526	CCTCTCCTCCAATAGCAGTCC	TCTCGGACTGCACGAAAACT	no
chr5:+113593900	GAACAGTAGAGGCTGGATTTTGA	TGTGTTAACCAGTCAGGGTGT	yes
chr5:+73647675	TTTCCCCAAACAGCACCTACC	CAATCAGGAAGGCGAGTGG	no
chr7:+4434126	GCTGGTGAGAGCAGAAGAGACC	GGGAGAATGAGCGACTTCGTG	no
chr2:-151931498	GCCACCAAGGAAGAGGTATGG	CCAGTCCGTCAAGGTCACAC	no

Supplementary Table 1: Potential off-targets analyzed after CRISPR. Loci were amplified around the potential off-target site with the shown forward and reverse primers, followed by PCR clean-up and sequencing of PCR products by using either forward or reverse primer.
