# Supplementary material for: Super-resolution microscopy compatible fluorescent probes reveal endogenous glucagon-like peptide-1 receptor distribution and dynamics
Source: Nat Commun. 2020 Jan 24;11:467. doi: 10.1038/s41467-020-14309-w (PMC6981144; doi:10.1038/s41467-020-14309-w)
Supplement: Supplementary file 3 — Description of Additional Supplementary Files [file 41467_2020_14309_MOESM3_ESM.pdf]

Description of Additional Supplementary Files

File name: Supplementary Movie 1

Description: Two-photon z-stack of **LUXendin645-**labeled islets (147 µm).

File name: Supplementary Movie 2

Description: Single-molecule localization microscopy in **LUXendin645-**labeled CHO-K1-SNAP_GLP1R cells (95 nm per pixel) (20 frames per second).

File name: Supplementary Movie 3

Description: Single-molecule localization microscopy in **LUXendin651-**labeled CHO-K1-SNAP_GLP1R cells (95 nm per pixel) (20 frames per second).

File name: Supplementary Movie 4

Description: Single particle tracking in **LUXendin651**-labeled CHO-K1-SNAP_GLP1R cells (47.5 nm per pixel) (20 frames per second).
