# Supplementary material for: Super-resolution microscopy compatible fluorescent probes reveal endogenous glucagon-like peptide-1 receptor distribution and dynamics
Source: Nat Commun. 2020 Jan 24;11:467. doi: 10.1038/s41467-020-14309-w (PMC6981144; doi:10.1038/s41467-020-14309-w)
Supplement: Supplementary file 8 — Reporting Summary [file 41467_2020_14309_MOESM8_ESM.pdf]

nature research

Corresponding author(s): David J. Hodson
Johannes Broichhagen
Tom Podewin

Last updated by author(s): 2019/12/19

Reporting Summary

Nature Research wishes to improve the reproducibility of the work that we publish. This form provides structure for consistency and transparency in reporting. For further information on Nature Research policies, see Authors & Referees and the Editorial Policy Checklist .

Statistics

For all statistical analyses, confirm that the following items are present in the figure legend, table legend, main text, or Methods section.

n/a	Confirmed	
☐	☒	The exact sample size (*n*) for each experimental group/condition, given as a discrete number and unit of measurement
☐	☒	A statement on whether measurements were taken from distinct samples or whether the same sample was measured repeatedly
☐	☒	The statistical test(s) used AND whether they are one- or two-sided<br>*Only common tests should be described solely by name; describe more complex techniques in the Methods section.*
☒	☐	A description of all covariates tested
☐	☒	A description of any assumptions or corrections, such as tests of normality and adjustment for multiple comparisons
☐	☒	A full description of the statistical parameters including central tendency (e.g. means) or other basic estimates (e.g. regression coefficient) AND variation (e.g. standard deviation) or associated estimates of uncertainty (e.g. confidence intervals)
☐	☒	For null hypothesis testing, the test statistic (e.g. *F*, *t*, *r*) with confidence intervals, effect sizes, degrees of freedom and *P* value noted<br>*Give P values as exact values whenever suitable.*
☒	☐	For Bayesian analysis, information on the choice of priors and Markov chain Monte Carlo settings
☒	☐	For hierarchical and complex designs, identification of the appropriate level for tests and full reporting of outcomes
☒	☐	Estimates of effect sizes (e.g. Cohen's *d*, Pearson's *r*), indicating how they were calculated

Our web collection on statistics for biologists contains articles on many of the points above.

Software and code

Policy information about availability of computer code

Data collection	cAMP and TIRF imaging data were acquired using Metamorph (Molecular Devices). Immunohistochemistry and live cell images were collected using Metamorph (Molecular Devices), Zen 2012 (Zeiss) and Leica Application Suite X (Leica).
Data analysis	Images were analyzed using ImageJ 1.5j8 (NIH), Zen 2012 (Zeiss) and Leica Application Suite X (Leica). Numerical data were analyzed using MATLAB R2018b (Mathworks) and Prism 8 (Graphpad).

For manuscripts utilizing custom algorithms or software that are central to the research but not yet described in published literature, software must be made available to editors/reviewers. We strongly encourage code deposition in a community repository (e.g. GitHub). See the Nature Research guidelines for submitting code & software for further information.

Data

Policy information about availability of data

All manuscripts must include a data availability statement. This statement should provide the following information, where applicable:

- Accession codes, unique identifiers, or web links for publicly available datasets
- A list of figures that have associated raw data
- A description of any restrictions on data availability

LUXendins are freely available for academic use. Glp1r(GE)-/- animals are subject to a Material Transfer Agreement. The source data underlying Figs 2a-c, g, k, 3c-f, 4f and h, 5e-g, 6c, 8b and d, and 9c-f and Supplementary Fig 1 are provided as a Source Data file.

Field-specific reporting

Please select the one below that is the best fit for your research. If you are not sure, read the appropriate sections before making your selection.

Life sciences study design

All studies must disclose on these points even when the disclosure is negative.

Sample size	The measurement unit is animal, batch of islets, well of cells or spheroid. Experiments were repeated independently at least three times, usually with technical replicates. Islet data are reported from at least two separate isolation procedures.
Data exclusions	No data were excluded unless the cells displayed a clear non-physiological state (i.e. impaired viability).
Replication	All findings were replicated independently three times, usually with different batches of compounds and across different investigators.
Randomization	Samples and animals were allocated to treatment groups in a randomized manner to ensure that all states were represented in the different experiment arms.
Blinding	Data were acquired using imaging setups that performed the measurement independently of the observer.

Reporting for specific materials, systems and methods

We require information from authors about some types of materials, experimental systems and methods used in many studies. Here, indicate whether each material, system or method listed is relevant to your study. If you are not sure if a list item applies to your research, read the appropriate section before selecting a response.

Materials & experimental systems

n/a	Involved in the study	
☐	☒	Antibodies
☐	☒	Eukaryotic cell lines
☒	☐	Palaeontology
☐	☒	Animals and other organisms
☐	☒	Human research participants
☒	☐	Clinical data

Methods

n/a	Involved in the study	
☒	☐	ChIP-seq
☒	☐	Flow cytometry
☒	☐	MRI-based neuroimaging

Antibodies

Antibodies used	GLP1R (DSHB Iowa, F6A11), INS (Cell Signaling, 3014), GLU (Sigma-Aldrich, G2654) and SST (Invitrogen, 14-9751-80).
Validation	Antibodies were validated using knockout tissue (GLP1R) or known cell-type specific localizations (INS, GLU and SST), further confirmed using reporter animals.

Eukaryotic cell lines

Policy information about cell lines

Cell line source(s)	AD293 (Agilent), CHO-K1-SNAP_GLP1R and HEK-SNAP_GLP1R (both Dr. Ben Jones, Imperial College London), SNAP_GLP1R_INS1GLP1R-/- (Dr. Jacqui Naylor, MedImmune).
Authentication	AD293 cells were authenticated at source. The other cell lines were authenticated using SNAP-labeling , antibiotic selection or lack of antibody staining.
Mycoplasma contamination	Mycoplasma testing was carried out throughout the facility once every 3 months, with all results negative.
Commonly misidentified lines (See ICLAC register)	No commonly misidentified lines were used.

Animals and other organisms

Policy information about studies involving animals; ARRIVE guidelines recommended for reporting animal research

Laboratory animals	Mouse, CD1, C57BL6, Glp1r(GE)-/-, Ins1CreThor;R26mT/mG, GLU-YFP, GLP1RCre;LSL-GCaMP3. Male and female, 6-12 wks.
Wild animals	No wild animals were used in the present study.
Field-collected samples	No field-collected samples were used in the present study.

Ethics oversight	Studies were approved by the UK Home Office, the University of Birmingham Animal Welfare and Ethics Review Board, the University College London Animal Welfare and Ethics Review Board, the University of Manchester Animal Welfare and Ethics Review Board and the Indiana University Institutional Animal Care and Use Committee (IACUC).

Note that full information on the approval of the study protocol must also be provided in the manuscript.

Human research participants

Policy information about studies involving human research participants

Population characteristics	Islets were obtained from brain-dead or deceased human donors with all necessary permissions and written informed consent from the family. WA01/H1 hESCs were obtained from WiCell.
Recruitment	No participants were recruited. Tissue was harvested from from brain-dead or deceased donors.
Ethics oversight	Procurement of human islets was approved by the Human Research Ethics Board (Pro00013094; Pro00001754) at the University of Alberta and all families of organ donors provided written informed consent. hESC (WA01/H1; hPSCreg name WAe001-A) (obtained from WiCell) were generated by the originating institute with informed consent and ethical approval from the Robert-Koch Institut, Berlin (Az.3.04.02/0101) and NIH (NIHhESC-10-0043). Studies with hESC (WA01/H1) were approved by the BC Children's and Women's Hospital Human Research Ethics Board (Approval #H09-00676). Studies with human tissue were approved by the BC Children's and Women's Hospital Human Research Ethics, University of Birmingham Ethics Committee and the National Research Ethics Committee (REC reference 16/NE/0107, Newcastle and North Tyneside, U.K.).

Note that full information on the approval of the study protocol must also be provided in the manuscript.
